# CAR T-cell Design-dependent Remodeling of the Brain Tumor Immune Microenvironment Modulates Tumor-associated Macrophages and Anti-glioma Activity

**DOI:** 10.1158/2767-9764.CRC-23-0424

**Published:** 2023-12-01

**Authors:** Dalia Haydar, Jorge Ibañez-Vega, Jeremy Chase Crawford, Ching-Heng Chou, Clifford S. Guy, Michaela Meehl, Zhongzhen Yi, Scott Perry, Jonathan Laxton, Trevor Cunningham, Deanna Langfitt, Peter Vogel, Christopher DeRenzo, Stephen Gottschalk, Martine F. Roussel, Paul G. Thomas, Giedre Krenciute

**Affiliations:** 1 St. Jude Children's Research Hospital, Department of Bone Marrow Transplantation and Cellular Therapy, Memphis, Tennessee.; 2 Children's National Hospital, Center for Cancer and Immunology Research, Washington, District of Columbia.; 3 Department of Immunology, St. Jude Children's Research Hospital, Memphis, Tennessee.; 4 Department of Microbiology Immunology Biochemistry, University of Tennessee Health Science Center, Memphis, Tennessee.; 5 Flow Cytometry Core, St. Jude Children's Research Hospital, Memphis, Tennessee.; 6 Department of Pathology, St. Jude Children's Research Hospital, Memphis, Tennessee.; 7 Department of Tumor Cell Biology, St. Jude Children's Research Hospital, Memphis, Tennessee.

## Abstract

Understanding the intricate dynamics between adoptively transferred immune cells and the brain tumor immune microenvironment (TIME) is crucial for the development of effective T cell–based immunotherapies. In this study, we investigated the influence of the TIME and chimeric antigen receptor (CAR) design on the anti-glioma activity of B7-H3–specific CAR T-cells. Using an immunocompetent glioma model, we evaluated a panel of seven fully murine B7-H3 CARs with variations in transmembrane, costimulatory, and activation domains. We then investigated changes in the TIME following CAR T-cell therapy using high-dimensional flow cytometry and single-cell RNA sequencing. Our results show that five out of six B7-H3 CARs with single costimulatory domains demonstrated robust functionality *in vitro*. However, these CARs had significantly varied levels of antitumor activity *in vivo*. To enhance therapeutic effectiveness and persistence, we incorporated 41BB and CD28 costimulation through transgenic expression of 41BBL on CD28-based CAR T-cells. This CAR design was associated with significantly improved anti-glioma efficacy *in vitro* but did not result in similar improvements *in vivo*. Analysis of the TIME revealed that CAR T-cell therapy influenced the composition of the TIME, with the recruitment and activation of distinct macrophage and endogenous T-cell subsets crucial for successful antitumor responses. Indeed, complete brain macrophage depletion using a CSF1R inhibitor abrogated CAR T-cell antitumor activity. In sum, our study highlights the critical role of CAR design and its modulation of the TIME in mediating the efficacy of adoptive immunotherapy for high-grade glioma.

**Significance:**

CAR T-cell immunotherapies hold great potential for treating brain cancers; however, they are hindered by a challenging immune environment that dampens their effectiveness. In this study, we show that the CAR design influences the makeup of the immune environment in brain tumors, underscoring the need to target specific immune components to improve CAR T-cell performance, and highlighting the significance of using models with functional immune systems to optimize this therapy.

## Introduction

High-grade gliomas are the deadliest primary brain tumors, with a median survival of 15 months (1). Current therapies including radiation, chemotherapy, or surgery are unable to cure the disease and are often associated with serious adverse effects (1). Immunotherapy using chimeric antigen receptor (CAR) T-cells offers a potentially safer and more effective alternative. CAR T-cells have shown promising results in preclinical brain tumor settings (2–4); however, they have shown limited success in clinical trials (5–8). The lack of success translating promising preclinical findings into viable clinical applications is in part due to the use of animal models that do not recapitulate human disease. Specifically, immunodeficient xenograft models do not represent the complex immune niche. Thus, studies using immunocompetent mouse models are needed to address our limited knowledge of the brain tumor immune microenvironment (TIME) during CAR T-cell treatment.

The brain TIME is highly complex and heterogeneous (9). Indeed, the TIME is dynamic and can change during tumor progression or in response to different therapeutic interventions (10, 11). However, mechanistic studies on how the TIME affects CAR T-cell functions remain limited. Understanding key factors within the TIME can lead to the identification of new therapeutic targets that may enhance CAR T-cell effector function.

Lessons learned from previous studies show that careful selection of CAR functional domains is essential for effective CAR T-cell antitumor response (3, 12–14). Published data suggest faster and better activation of CD28-CARs, while 41BB-CARs are associated with improved overall persistence (15). CD28-CARs signal through the PI3K-Akt pathway, whereas 41BB-CARs signal through the recruitment of TRAF proteins (15). In addition, calibrating CAR activation domains through inactivation of the first and third immunoreceptor tyrosine-based activation motifs (ITAM) of CD3ζ results in decreased apoptosis and nonspecific activation of CAR T-cells (16). Although the differential impact of CAR domains has been well established (17–19), there are no studies evaluating the impact of different CAR functional domains on the composition and function of the TIME.

To address these gaps in knowledge, we generated a panel of murine (m) B7-H3–specific CARs containing different transmembrane, costimulatory, and activation domains and compared their effector function *in vitro* and *in vivo*. We show that five out of six B7-H3-CARs with varying transmembrane, costimulatory, and activation domains, exhibit robust functionality *in vitro*. Interestingly, incorporating 41BBL (3, 20) signaling into our prototype 28.*m*ζ CAR T-cells (21) significantly enhanced their expansion and persistence *in vitro*. However, these highly efficacious CAR T-cells showed poor antitumor responses *in vivo*. Single-cell RNA sequencing (scRNA-seq) analyses revealed distinctive changes in the TIME in response to different CAR constructs. We found immune cell clusters containing macrophages coexpressing both M1 and M2 genes to be associated with potent CAR T-cell responses. Indeed, global macrophage depletion using CSF1R inhibitors completely abrogated CAR T-cell therapeutic effects. Together, we show that successful tumor control by CAR T-cells is dependent on CAR structural design and requires functional T cells and a balance of proinflammatory and anti-inflammatory macrophages to prevent treatment failure and rapid CAR T-cell exhaustion.

## Materials and Methods

### Cell Lines

The murine GL261 glioma cell line was procured from the Leibniz Institute (DSMZ-German Collection of Microorganisms and Cell Cultures). The human embryonic kidney cell line 293T (also known as HEK 293T, RRID: CVCL_0063) and the murine embryonic fibroblast packaging cells GPE-86 (CRL-9642) were obtained from ATCC. To create the *B7-h3* knockout GL261 (GL261-KO) cell line, CRISPR-Cas9 technology was employed, as detailed in ref. 21.

GL261 murine tumor cells were cultured in complete RPMI media as described in ref. 21 (briefly RPMI media was supplemented with 10% FBS, 1% glutamax, 1% penicillin/streptomycin, 1% sodium pyruvate, 1% MEM non-essential amino acids, and 0.1% 2-mercaptoethanol). GPE86 and 293T cells were maintained in DMEM with 10% FBS, 1% glutamax, and 1% penicillin/streptomycin. In addition, murine T cells were cultured in complete RPMI media (supplemented with 10% FBS, 1% glutamax, 1% penicillin/streptomycin, 1% MEM non-essential amino acids, and 0.1% 2-mercaptoethanol).

*Mycoplasma* screening was performed every 3–4 months using the MycoAlert Mycoplasma Detection Kit from Lonza. The cell lines were maintained for a maximum of 2 months (or 12 passages) in culture after which new cells from lower passages were thawed. Authentication of all cell lines was performed by IDEXX Bioanalytics.

### Animal Models

All animal experiments adhered to the approved protocol (#623-100650) established by the St. Jude Children's Research Hospital Institutional Animal Care and Use Committee. Mice were humanely euthanized when they exhibited physical euthanasia criteria such as significant weight loss or signs of distress, or when advised by the veterinary staff at St. Jude.

Albino C57BL/6 mice [B6(Cg)-Tyrc-2J/J, 000058, The Jackson Laboratory] were acquired at 6–8 weeks of age. All animals utilized for tumor implantation were males or females ages 10–12 weeks. For intracranial implantation, mice were anesthetized and secured in a stereotactic rodent surgery platform. A 1-mm burr hole was carefully created in the skull at a point 1 mm anterior and 2 mm to the right of the bregma. Subsequently, 1 × 10^5^ GL261 cells suspended in 2 µL of raw media were injected to a depth of 3 mm, following the procedure outlined in ref. 21. The surgical site was then closed using wound clips.

On day 7 after tumor implantation, animals were treated with 3 × 10^6^ effector cells, adjusted to achieve 40% CAR expression, and suspended in 2 µL of media through intracranial injection, using the same burr hole coordinates used for tumor implantation. Each experimental group consisted of 5 mice. Experiments were repeated with CAR T-cells derived from distinct biological donors.

For experiments with BLZ945 combination, oral treatment was started on day 5 after tumor implantation or at 4 days prior. Briefly, BLZ945 (TargetMol) was resuspended in 20% Captisol (Captisol) at 60 mg/mL. Mice were dosed at 200 mg/kg in 100 µL via oral gavage. Control treated mice received 20% Captisol. Treatment continued daily for 3–4 weeks, and fresh drug formulation was prepared every other day for the duration of treatment.

### Generation of Murine CAR Vectors

To generate mB7-H3 CAR T-cells, we designed retroviral vectors encoding second-generation CARs with an antigen recognition domain derived from the mB7-H3–specific monoclonal antibody m276. We cloned six constructs: (i) ^CD28tm^CD28.ζ; (ii)^CD28tm^41BB.ζ; (iii) ^CD8tm^41BB.ζ; and (iv–vi) same as (i–iii) with two additional mutations in the first and third ITAMs of the CD3ζ signaling domain as described in ref. 16. As a control, we cloned a nonfunctional mB7-H3 CAR without a signaling domain. Finally, to generate CD28-based CARs expressing 41BBL on the T-cell surface, we cloned a vector encoding murine 41BBL, a 2A sequence, and the CD28-CAR with mutated CD3ζ domain. All final CAR vectors were verified by sequencing.

### Generation of Retroviral Particles for Murine T-cell Transduction

Retroviral particles used for the transduction of murine T cells were produced from GPE-86 producer cell lines stably expressing each of the eight different CARs which were generated as detailed in ref. 21. In brief, transient viral supernatant was initially generated by transfecting HEK293T cells with the CAR-encoding vector (in addition to Peq-Pam and VSVG plasmids). Subsequently, the GPE-86 producer cell lines were generating by transduction with the pseudotyped viral particles from 293T cells in combination with polybrene (8 mg/mL, Millipore Sigma). GPE-86 cells were then labeled for CAR expression using AF-647 anti-human IgG (F(abʹ)2 fragment-specific) and sorted using FACS (BD FACSAria III) to enrich for the CAR-positive cells.

For each CAR T-cell transduction experiment, fresh supernatant from the relevant GPE-86 producer cell lines was generated by maintaining the sorted producer cell lines in culture using Iscove's modified Dulbecco's medium supplemented with 10% FBS and 1% glutamax.

### Generation of Murine CAR T-cells

mB7-H3 CAR T-cells were generated through the standard process of retroviral transduction of murine T cells, as outlined in ref. 21. Murine T cells were selectively purified from mouse spleens using the CD3^+^ T-Cell Isolation Kit (Miltenyi Biotec). Spleens were obtained from 6–8 weeks old B6 Thy1.1 mice (000406, The Jackson Laboratory) and homogenized through a 70-µm cell strainer followed by lysis of red blood cells. CD3^+^ murine T cells were isolated following the bead selection process as detailed by the manufacturer's instructions (130-095-130, Miltenyi Biotec). Purified T cells were then activated using CD3/CD28 antibodies (553057 and 553294, respectively, BD, RRID: AB_10947764) in complete T-cell media with 50 U/mL of human IL2 (Peprotech).

Following a 48-hour activation period, T cells were transduced with GPE-86–derived retroviral particles, using RetroNectin-coated wells (Clontech). Subsequently, CAR T-cells were harvested 48 hours later and underwent expansion in the presence of IL2 (50 U/mL), IL7 (10 ng/mL), and IL15 (5 ng/mL; Peprotech) until day 7 after transduction. The detection of CAR expression was performed on days 3–5 using flow cytometry. Both *in vitro* and *in vivo* experiments were conducted either on or before day 9 after transduction.

### Functional Analysis of Murine CAR T-cells *In Vitro*

#### Serial Coculture Assay

To perform repeated stimulation assays, 5 × 10^5^ tumor cells (either GL261 or GL261-KO) were seeded in a 24-well plate and allowed to attach to the bottom of the well for 4–6 hours at 37°C. CAR T-cells were then washed with cytokine-free media, followed by adjusting CAR expression to 40% by adding non-transduced (NT) T cells to the CAR products that initially displayed CAR expression above 40%. Subsequently, 1 × 10^6^ T cells were introduced into the wells containing the tumor cells. After 3–4 days of coculture, CAR T-cells were collected and the T-cell count was determined using a hemocytometer. For consecutive stimulations, fresh tumor cells were seeded and maintained in culture with T cells at the same effector to target (E:T) ratio. These stimulations were repeated every 3–4 days until T cells ceased killing and/or expanding.

In experiments involving BLZ945 treatment, a drug stock (953769-46-5, AbMole) with a concentration of 10 mmol/L in DMSO was prepared and its purity and identity were verified through nuclear magnetic resonance and LC/MS. Working concentrations were then prepared in complete media and added directly after the coculture setup to achieve the desired final concentrations of 67, 670, and 6,700 nmol/L. Control wells were treated with equivalent concentrations of media containing DMSO.

#### Cytotoxicity Assay

To assess the cytotoxicity of CAR T-cells, a colorimetric MTS reagent [3-(4,5-dimethylthiazol-2-yl)-5-(3-carboxymethoxyphenyl)-2-(4-sulfophenyl)-2H-tetrazolium] from Promega was according to the previously described method (21). Tumor cells (GL261 or GL261-KO) were seeded in 96-well plates and allowed to settle and adhere for 4–6 hours. CAR T-cells were then washed to remove exogenous cytokines, adjusted to 40% CAR expression, and added to the wells containing tumor cells at various E:T ratios (4:1, 2:1, 1:1, 0.5:1, and 0.25:1). The cocultures were then incubated for 72 hours at 37°C. Experiments involving BLZ945 treatment utilized media prepared with the working drug concentrations as described previously.

After 3 days incubation, T cells were gently detached by pipetting, and the MTS reagent was diluted in complete media and added to each well. The mixture was then incubated at 37°C for 2–3 hours, and the absorbance was measured at 492 nm using an Infinite 200 Pro MPlex plate reader (Tecan). The percentage of live tumor cells was calculated using the following formula: [(Absorbance of the sample − Absorbance of media only)/(Absorbance of tumor only − Absorbance of media only)] × 100.

#### Cytokine Production

During each cycle of the repeated-stimulation assay, the supernatant was gathered 24 hours after coculturing T cells with tumor cells and promptly frozen at −80°C. The evaluation of cytokine production was carried out using a 32-plex murine cytokine quantification kit (MCYTMAG-70K-PX32, Millipore Sigma), and the analysis was conducted using a Luminex FlexMap three-dimensional instrument and its associated software (Luminex Corporation).

### Flow Cytometry

All basic flow cytometry data were generated using a BD FACSCanto II instrument and subsequently analyzed with FlowJo software (FlowJo, RRID:SCR_008520). Samples were washed and stained in FACS buffer (PBS supplemented with 1% FBS). In all experiments, matched isotypes or known negatives (NT T cells) were employed as gating controls. In addition, the Invitrogen Fixable Aqua Dead Cell Stain Kit (L34957, Thermo Fisher Scientific) was utilized as a viability dye.

For a comprehensive analysis of the TIME, flow cytometry data were acquired using a BD FACSymphony A5 cytometer (BD). This cytometer was equipped with 355, 400, 440, 488, 561, and 640 nm lasers and incorporated 30 fluorescent detectors. These detectors were equipped with long-pass and band-pass filters tailored to the specific experimental fluorochromes being used.

#### T Cells and Tumor Cell Lines

To examine B7-H3 expression on tumor cell lines, the cells were subjected to staining with CD276-AF647 (562862, clone MIH32, BD). For the phenotyping of T cells, the cells underwent staining with CD4-APC-Cy7 (552051, BD), CD8-FITC (553030, BD), CD62L-PerCpCy5.5 (560513, BD), and CD44-PE (553134, BD). The B7-H3-CAR was detected using AF647-anti-human IgG, F(abʹ)2 fragment-specific antibody (109-606-006, Jackson ImmunoResearch).

#### TIME Analysis

Brain tumor samples were isolated following the humane euthanasia of mice via CO_2_ inhalation, followed by cardiac perfusion with PBS. The cardiac perfusion with PBS was executed gradually to effectively clear blood vessels while preserving the brain and immune cells for subsequent analyses. Subsequently, the brains were extracted from the skulls, and the upper right quadrants of each brain, encompassing both the tumor and surrounding normal tissue, were gathered in 2 mL of RPMI supplemented with 5% FBS. The brain tumor samples were then finely minced using scissors and incubated with 1 mg/mL of collagenase IV (STEMCELL) and 50 U/mL of DNase I (Thermo Fisher Scientific) for 1 hour at 37°C.

Following enzymatic digestion, the samples were passed through a 70 µm cell strainer using a syringe plunger to achieve a single-cell suspension. The resulting cell suspension was then centrifuged, and the pellet was resuspended in 10 mL of RPMI with 5% FBS. Cell counts were determined, and the cells were subsequently aliquoted into FACS tubes for processing in preparation for flow cytometry analysis.

The initial step involved labeling the samples with Fixable Blue dead cell stain (Thermo Fisher Scientific) in accordance with the manufacturer's instructions. Subsequently, the samples were blocked to minimize nonspecific antibody binding by incubating them with an excess of purified IgG (Fc Block; BioLegend). Following a washing step, separate portions of the cells were resuspended in either lymphoid (19-antibody) or myeloid (21-antibody) optimized staining cocktails, as specified in Supplementary Table S1. The cells were typically allowed to incubate for 20 minutes on ice, followed by a washing step and resuspension in a minimal volume of staining buffer for subsequent flow cytometric analysis.

For cytokine expression profiling of tumor infiltrates, cell samples were first stimulated in culture with optimized concentrations of phorbol 12-myristate 13-acetate and ionomycin in the presence of monensin for 4 hours (Cell Activation Cocktail with Brefeldin A; BioLegend). The stimulated cell suspensions were then washed with PBS, labeled with Live/Dead Blue, and subjected to the blocking procedure involving excess IgG, as described above for surface immunophenotyping. These samples were subsequently incubated with the 11-antibody extracellular staining cocktail outlined in ST1 for cytokine expression profiling, followed by washing, fixation, and permeabilization using the eBioscience Foxp3/transcription factor staining set (Thermo Fisher Scientific) in accordance with the manufacturer's instructions. Once fixed and permeabilized, the cells were incubated with the 9-antibody cocktail for intracellular staining (ST1), washed, and resuspended in a minimal volume of staining buffer for flow cytometric analysis.

While conventional bivariate analyses were performed using FACS Diva software for standard cell surface markers, as indicated in Supplementary Table S2 (BD Biosciences), a more comprehensive understanding of marker expression patterns in the tumor infiltrates was obtained through the application of t-distributed stochastic neighbor embedding (t-SNE) algorithms, facilitated by FlowJo software (BD Biosciences).

### scRNA-seq

Brain tumors were obtained after transcardiac perfusion of mice with PBS, and then processed for FACS using the previously described enzymatic digestion method. The single cells were subsequently enumerated and stained in PBS containing 5% FBS. In brief, vials of TotalSeq-B antibodies were centrifuged and resuspended in 50 µL of staining buffer. Initially, the cells were incubated with Fc Block (BioLegend) for 10 minutes at 4°C. Following this, TotalSeq-B antibodies and flow cytometry antibodies (CD45-PE, Ter119-APC) were introduced, and the cells were incubated for 30 minutes at 4°C. This was followed by a washing step using staining buffer. Prior to sorting, DAPI was employed for staining dead cells. Two distinct populations, CD45^−^ TER119^−^ and CD45^+^ TER119^−^, were sorted into RPMI media with 10% FBS. The viable CD45^+^ and CD45^−^ populations were mixed at a ratio of 70:30 and processed using the Chromium system (10X Genomics, 3′ v3), targeting 10,000 cells per reaction. Gene expression and cell surface libraries were prepared in accordance with the 10x manufacturer's protocols, and subsequent sequencing was carried out on the Illumina NovaSeq 6000 platform.

### Bioinformatic Analyses

#### Raw Data Processing and Data Visualization

Raw sequencing data were processed using CellRanger (10X Genomics, v6.1.2, RRID:SCR_021002) with the corresponding mm10-2020-A reference. Individual reactions were then aggregated for read-normalization resulting in a mean of 43,828 post-normalized reads per cell. Downstream analyses were conducted with Seurat (v4.2.0; ref. 22) in R, retaining only genes found in at least 50 of the 42,105 post-aggregation cells. Dead and dying cells were excluded by removing those with at least 10% of gene expression owed to mitochondrial genes, and cells with fewer than 300 genes detected were likewise excluded. To remove putative multiplets, we excluded cells from each reaction that exhibited detected genes or RNA molecules at or above the 98% quantile for that respective reaction. Filtered expression data were subsequently normalized with default parameters. For clustering and dimensionality reduction, variable features were identified using the “vst” method, data were scaled without regression of other variables, and the top 1,000–3,000 variable genes were utilized for principal component analysis depending on the subset of data being analyzed. For macrophages and whole sample cluster analyses 1,000 genes were used, and 3,000 genes for T-cell clusters. Uniform Manifold Approximation and Projection (UMAP) dimensionality reduction and clustering utilized the first 7–30 principal components, depending upon the subset of the data under analysis.

To identify the cell identity of each cluster, we used the R package SingleR (23, 24), coupled to cellDex (23) using the Immunological Genome Project (25). Then, all the cell identities were corroborated on the basis of expression of the reported/canonical markers.

Differentially upregulated genes were performed using Seurat package, and for gene set enrichment analysis (GSEA), we utilized the Molecular Signature Database (MSigDB) by using the MSigDBr package in R (26). All terms with a *P* < 0.05 were considered significant and ranked by the number of genes identified in the group.

To infer cell–cell interaction in the scRNA-seq data, we used the R package CellChat (27). Briefly, we used the mouse ligand-receptor database, and inferred the cell-cell communication at the signal pathway level, to further aggregate the cell-cell communication. The most influential cell cluster was considered as the cluster that has the highest values in interaction as a sender/source.

To detect evidence of CAR transcripts, a *post hoc* analysis was performed by creating a custom reference comprised of mm10-2020-A and 729 bases of sequence from the CAR vector. Raw sequencing data were reprocessed with CellRanger v7.1.0 using this custom reference, and the resulting CAR UMI counts were extracted from the raw feature barcode matrices without read normalization and integrated into the Seurat objects used for previous analyses.

### MRI

Mice underwent imaging conducted by the St. Jude Center for In Vivo Imaging and Therapeutics (CIVIT). MRI was carried out using a Bruker Biospec 94/30 MRI system (Bruker Biospin MRI GmbH). Prior to the imaging session, the mice were anesthetized in a chamber with 3% isoflurane in oxygen delivered at a rate of 0.5 L/minute, and this anesthesia level was subsequently maintained through nose-cone delivery, with a mixture of 1%–2% isoflurane in oxygen, delivered at the same flow rate. To ensure the animals’ well-being and stability, a heated bed with warm water circulation and a physiologic monitoring system were employed to monitor the respiratory rate.

During the MRI procedure, a mouse brain surface receive coil was positioned over the mouse's head and placed within an 86 mm transmit/receive coil. Following the initial localization step, T2-weighted Rapid Acquisition with Refocused Echoes (RARE) sequences were conducted in both the coronal (TR/TE = 2,000/20.4 ms, matrix size = 256 × 256, field of view = 20 mm × 20 mm, slice thickness = 0.5 mm, number of slices = 16) and axial (TR/TE = 2500/23 ms, matrix size = 256 × 256, field of view = 20 mm × 20 mm, slice thickness = 0.5 mm, number of slices = 32) orientations.

### IHC and H-score Determination

The brains were subjected to perfusion and fixation in 10% neutral buffered formalin, followed by embedding in paraffin. These paraffin-embedded specimens were sectioned into 5 mm slices, mounted on positively charged glass slides (Superfrost Plus, Thermo Fisher Scientific), and then dried at 60°C for 20 minutes. Subsequently, the sections were dewaxed and stained with hematoxylin and eosin using standard techniques.

For IHC detection of CD276 (B7-H3), CD3, and IBA1, the tissue sections underwent antigen retrieval using prediluted Cell Conditioning Solution (CC1; Ventana Medical Systems) for 30 minutes. In the case of F4/80 and CD11c, antigen retrieval utilized Epitope Retrieval solution 1 (ER1) for 20 minutes and Epitope Retrieval solution 2 (ER2) for 30 minutes at 100°C, both performed on a Bond Max immunostainer (Leica Biosystems). The primary antibody for detecting CD276 (B7-H3) was diluted at 1:200 (AF1027, R&D Systems) and paired with the OmniMap anti-rabbit HRP kit (Ventana Medical Systems). To identify macrophages, two different antibodies were employed: anti-IBA1 (1:300 dilution, CP290A; Biocare Medical) and rabbit anti-F4/80 (1:750, 70076; Cell Signaling Technology, RRID:AB_10372054). Additional antibodies included anti-CD3 for T-cell detection (1:500 dilution, sc-1127; Santa Cruz Biotechnology) and CD11c (1:200 dilution, 97585, Cell Signaling Technology, RRID:AB_2800282).

H-scores were calculated using the HALO automated image analysis program (v3. 2.1851. 3, Indica Labs) based on whole-slide digital images to evaluate the intensity and extent of CD276 (B7-H3)-specific staining within the tumors. In this process, the HALO cytonuclear image analysis algorithm was initially optimized and subsequently applied using tissue classifiers and annotations to quantify the percentage of cancer cells displaying strong (3+), moderate (2+), weak (1+), or negative staining. These measurements were used to calculate CD276 (B7-H3) H-scores for each sample. All sections were reviewed by a pathologist who was unaware of the experimental group assignments.

### Data Availability

Raw unprocessed sequencing data for single-cell expression experiments have been archived in the Sequence Read Archive under the BioProject Accession PRJNA955817.

### Statistical Analyses

All experiments were conducted with a minimum of two replicates. When comparing two groups, a two-tailed *t* test was employed. In cases involving three or more groups with a single independent variable, statistical significance was assessed using one-way ANOVA along with Tukey multiple comparisons test. For comparisons among three or more groups with two or more independent variables, the statistical significance was determined through multiple *t* tests or two-way ANOVA accompanied by Sidak or Tukey multiple comparisons test.

Survival curves were generated utilizing the Kaplan–Meier method, and the statistical significance between survival curves was evaluated using the log-rank (Mantel–Cox) test. The analysis of bioluminescence imaging data was carried out employing either ANOVA or a *t* test. *P* values were calculated using Prism software (GraphPad Software, RRID:SCR_002798).

Statistical significance is represented as follows: *, *P* < 0.05; **, *P* < 0.01; ***, *P* < 0.001; ****, *P* < 0.0001; ns, nonsignificant. To simplify figures involving multiple group comparisons, differences in groups that did not demonstrate statistical significance are not displayed.

## Results

### mB7-H3 CAR T-cells Have Limited Anti-glioma Activity in Immunocompetent Models

We previously evaluated B7-H3 CAR T-cell therapy in an immunocompetent glioma model, where we demonstrated a significant survival advantage without signs of toxicity (21). Following intratumoral injection of mB7-H3 CAR T-cells (28.*m*ζ) into C57BL/6 mice transplanted with GL261 tumor cells, only 60% of treated mice had complete responses (Supplementary Fig. S1A and S1D). The limited therapeutic responses were not associated with antigen downregulation, as evaluated by mB7-H3 IHC staining from nonresponder mice (Supplementary Fig. S1E–S1F). Thus, we first sought to improve these CAR T-cells by optimizing CAR structure.

### Design and Expression of mB7-H3 CARs with Different Domains

**FIGURE 1:**
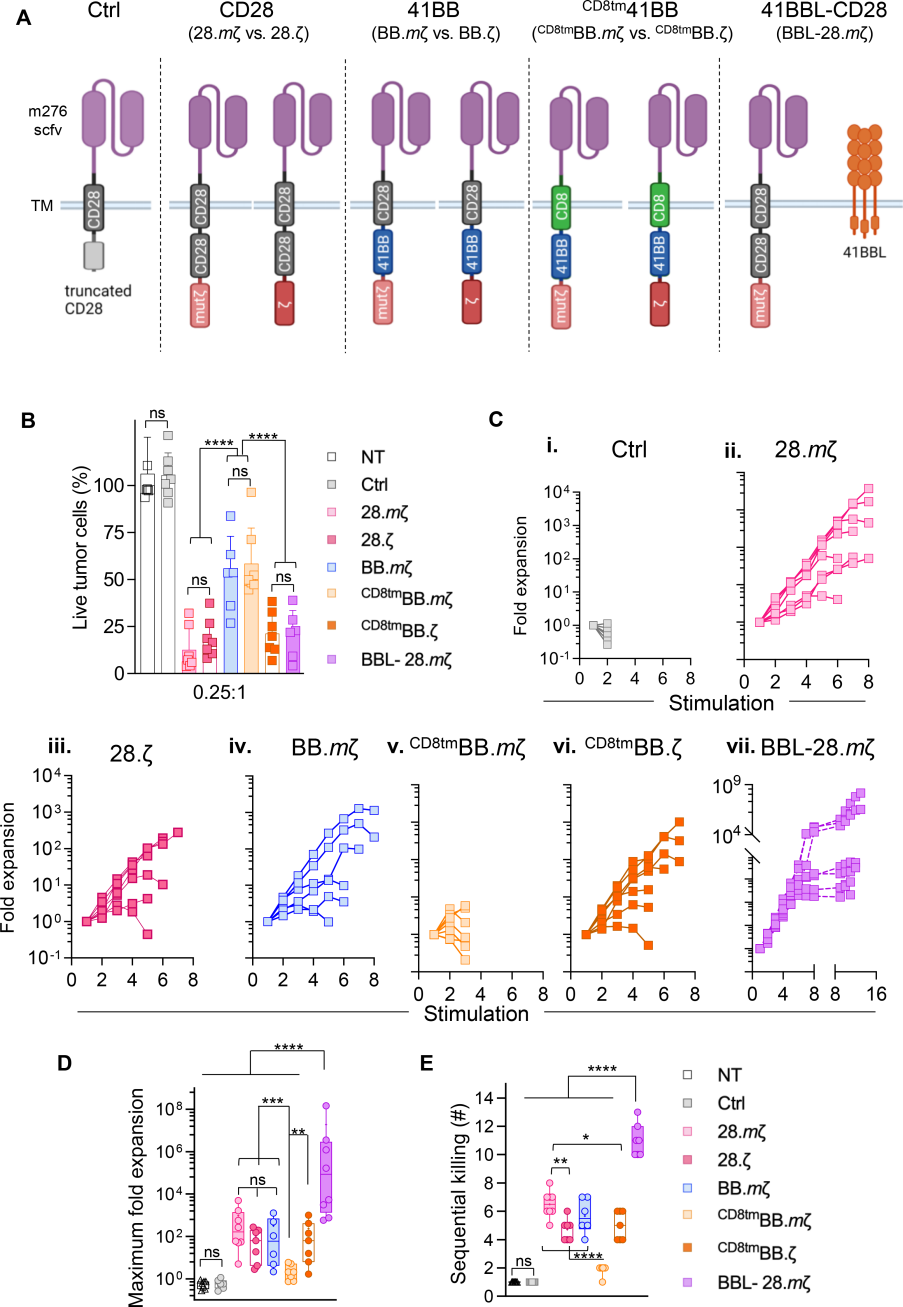
Generation and functional characterization of syngeneic B7-H3 CAR T-cells with different CAR structural designs. **A,** Scheme of mB7-H3-CAR constructs. Created with BioRender.com. **B,** MTS cytotoxicity assay against GL261 tumor cells at an E:T ratio of 0.25:1 (*n* = 7, mean ± SD, two-way ANOVA with Tukey test for multiple comparisons). **C,** T cells expressing different mB7-H3 CAR constructs were cocultured with GL261 tumor cells at a 2:1 ratio with restimulation every 3 days against fresh tumor cells until they no longer killed and/or expanded. Graphs show fold expansion of different T-cell donors upon successive stimulations (*x*-axis: each stimulation is a 3-day coculture with fresh GL261 tumor cells, *n* = 7). **D,** Summary of the maximum fold expansion of mB7-H3 CAR T-cells from individual donors upon repeat stimulation with GL261 tumor cells (*n* = 7, minimum to maximum range, one-way ANOVA with Tukey test for multiple comparisons). **E,** Maximum number of times CAR T-cells were able to kill Gl261 tumor cells (*n* = 7, minimum to maximum range, one-way ANOVA with Tukey test for multiple comparisons).

To investigate the role of structural CAR design in immunocompetent glioma models, we generated a panel of mB7-H3 CARs containing different transmembrane, costimulatory, and activation domains (Fig. 1A; Supplementary Fig. S2). Our initial studies (21) included mB7-H3 CARs with CD28 transmembrane and costimulatory domains with mutated CD3ζ activation domain (**28.*m*ζ**). To first evaluate the effect of activation domains on anti-glioma activity of mB7-H3 CAR T-cells, we cloned a CD28-CAR with intact CD3ζ (**28.ζ**). As a control, we used an mB7-H3 CAR with a truncated endodomain (**Ctrl**). In addition, we cloned two CARs with CD28 transmembrane domains and 41BB costimulatory domains, with a mutated or intact CD3ζ (**BB.*m*ζ** and **BB.ζ**). Similarly, we cloned two additional 41BB-CARs with a CD8 transmembrane domain (**^CD8tm^BB.*m*ζ** and **^CD8tm^BB.ζ**). Finally, we designed a CAR incorporating 41BB and CD28 costimulation through transgenic expression of 41BBL on the surface 28.*m*ζ CAR T-cells (**BBL-28.*m*ζ**).

CAR T-cells were generated by standard retroviral transduction as described previously (21). We observed variable CAR expression on the surface of transduced murine T cells despite efficient expression of the eight described constructs on GPE86 producer cell lines (Supplementary Fig. S3A and S3B). Moreover, the BB.ζ-CAR was consistently expressed at low levels on the T-cell surface and intracellularly (Supplementary Fig. S3C, Surface: 17.95 ± 2.46; Intracellular: 22.1± 1.41). Thus, this construct was excluded from additional testing. To ensure that differences in CAR expression did not confound our results, NT T cells were added to each product to achieve a final CAR-positive level of 40% (Supplementary Fig. S3D, *P* < 0.0001). All CAR T-cells were used immediately after CAR-positive level adjustment (Supplementary Fig. S3E).

### Functional Characterization of mB7-H3-CARs with Different Domains

We next tested mB7-H3 CAR T-cell memory phenotype. We observed a comparable ratio of CD4 to CD8 T cells among all constructs except for Ctrl-CAR, which had slightly higher CD4/CD8 ratio (Supplementary Fig. S4A, *P* < 0.05). There were no significant differences in T-cell memory phenotypes for CAR-positive cells except for the BBL-28.*m*ζ transduced T cells, which had a slightly higher proportion of central memory phenotype (Supplementary Fig. S4B, *P* < 0.05).

To evaluate whether different CAR domains can alter B7-H3 CAR T-cell cytotoxicity, we performed a 72-hour MTS assay. Cells were cocultured at different E:T ratios without any exogenous cytokines. Cytolysis was then measured by colorimetric quantification of viable tumor cells. In contrast to controls (NT or Ctrl-CAR), all mB7-H3 CARs had efficient cytolytic activity against GL261 tumor cells at higher E:T ratios (Supplementary Fig. S4C, *P* < 0.0001). However, 41BB-based CARs with mutated CD3ζ (BB.*m*ζ and ^CD8tm^BB.*m*ζ) exhibited significantly reduced cytolytic activity at lower E:T ratios (Fig. 1B, NT and Ctrl vs. BB.*m*ζ or ^CD8tm^BB.*m*ζ *P* < 0.0001; BB.*m*ζ vs. ^CD8tm^BB.*m*ζ *P* > 0.05; BB.*m*ζ or ^CD8tm^BB.*m*ζ vs. other constructs *P* < 0.0001). Importantly, all CARs exhibited no cytotoxicity against B7-H3-negative target cells confirming specificity (GL261-KO; Supplementary Fig. S4D).

### Surface Expression of 41BBL Provides Expansion and Persistence Advantages

To evaluate whether altering CAR domains can enhance the sequential killing and persistence of mB7-H3 CAR T-cells, T cells expressing different mB7-H3 CAR constructs were cocultured with GL261 or GL261-KO at an E:T ratio of 2:1 without any exogenous cytokines. If T cells killed and expanded, they were restimulated with fresh tumor cells every 3 days. All mB7-H3 CAR T-cells killed and expanded for up to two stimulations against GL261 tumor cells except for NT and Ctrl CAR T-cells (Fig. 1C). There were no significant differences in the maximum fold expansion among CARs with single costimulatory domains except for the ^CD8tm^BB.*m*ζ-CAR, which failed to expand or persist beyond two stimulations (Fig. 1D, ^CD8tm^BB.*m*ζ vs. NT or Ctrl *P* > 0.05; ^CD8tm^BB.*m*ζ vs. other constructs *P* < 0.05). Our prototype CAR (28.*m*ζ) had enhanced persistence averaging about 3 weeks in repeat-stimulation assays (6–7 sequential killings) versus 0.7–2 weeks for other constructs (2–5 sequential killings; Fig. 1E, 28.*m*ζ vs. ^CD8tm^BB.ζ *P* < 0.05; 28.*m*ζ vs. other constructs *P* < 0.005). Remarkably, transgenic expression of 41BBL on the surface of 28.*m*ζ CAR T-cells provided a significant expansion and persistence advantage when compared with all other CAR T-cells (Fig. 1C_vii_ and Fig. 1D–E, BBL-28.*m*ζ vs. other constructs *P* < 0.0001). Moreover, expansion was antigen-specific with no expansion or persistence upon stimulation against GL261-KO or media only (Supplementary Fig. S5).

### mB7-H3 CAR T-cells Show Sustained Cytokine Secretion Upon Multiple Stimulations

**FIGURE 2:**
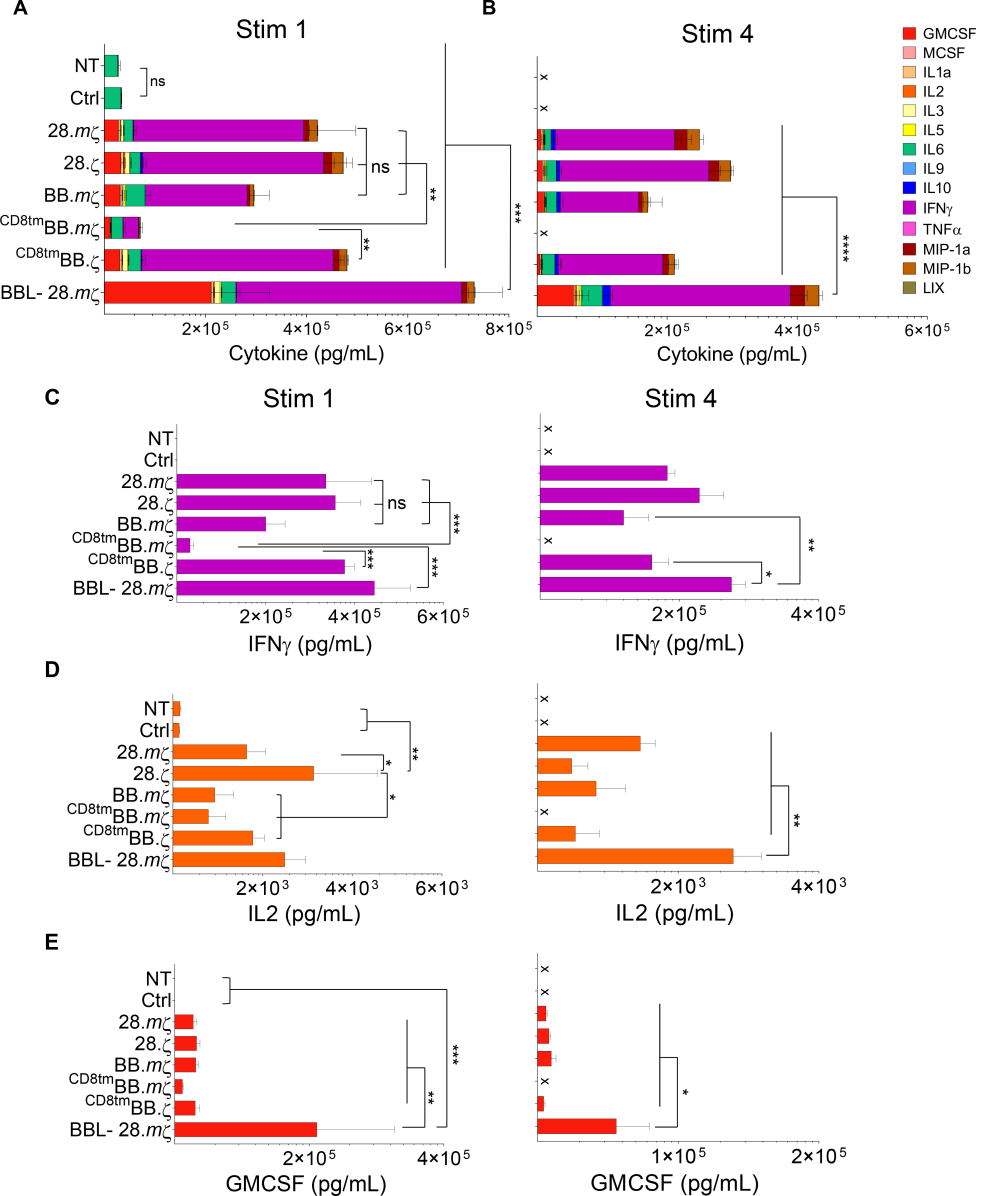
Surface expression of 41BBL on CD28-based mB7-H3-CAR T-cells enhances effector cytokines release in repeat stimulation assay. Culture supernatants were collected at 24 hours after repeated stimulation with GL261 tumor cells at 2:1 ratio and analyzed using Multiplex assay. Summary plots of cytokines and chemokines produced by CAR T-cells after first stimulation (**A**) and fourth stimulation (**B**) against GL261 tumor cells (*n* = 4, mean ± SEM, two-way ANOVA with Tukey test for multiple comparisons). **C–E,** CAR T-cell production of IFNγ, IL2, and GMCSF at 24 hours’ after stimulations one and four (*n* = 4, mean ± SEM, two-way ANOVA with Tukey test for multiple comparisons).

To evaluate the effector functions of different mB7-H3 CARs, we measured cytokine secretion upon repeated-stimulation. Coculture supernatants were collected at 24 hours after each stimulation, and the concentrations of Th1 (IFNγ, IL2, TNFα, IL1α, GMCSF, IL3) and Th2 (IL4, IL5, IL6, IL9, IL10) cytokines and chemokines (LIX, MIP-1a, MIP-1b) were measured using a multiplex assay. Compared with NT or Ctrl CAR T-cells, all mB7-H3 CARs secreted significantly higher levels of cytokines after stimulation with GL261 except for ^CD8tm^BB.*m*ζ-CAR, which is consistent with the expansion data (Fig. 2A, stim 1: NT and Ctrl vs. ^CD8tm^BB.*m*ζ *P* > 0.05, NT and Ctrl vs. other constructs *P* < 0.005). Importantly, BBL-28.*m*ζ CAR T-cells produced significantly higher levels of cytokines compared with T cells transduced with other constructs (Fig. 2A, stim 1: BBL-28.*m*ζ vs. other constructs *P* < 0.005). With subsequent stimulations, BBL-28.*m*ζ CAR sustained higher levels of cytokine production (Fig. 2B, stim 4: BBL-28.*m*ζ vs. other constructs *P* < 0.0001). Specifically, BBL-28.*m*ζ CAR T-cells released higher levels of IFNγ, IL2, and GMCSF at first and fourth stimulations with GL261 tumor cells (Fig. 2C–E, BBL-28.*m*ζ vs. other constructs *P* < 0.05).

### *In Vitro* Findings do not Accurately Predict *In Vivo* Performance

**FIGURE 3:**
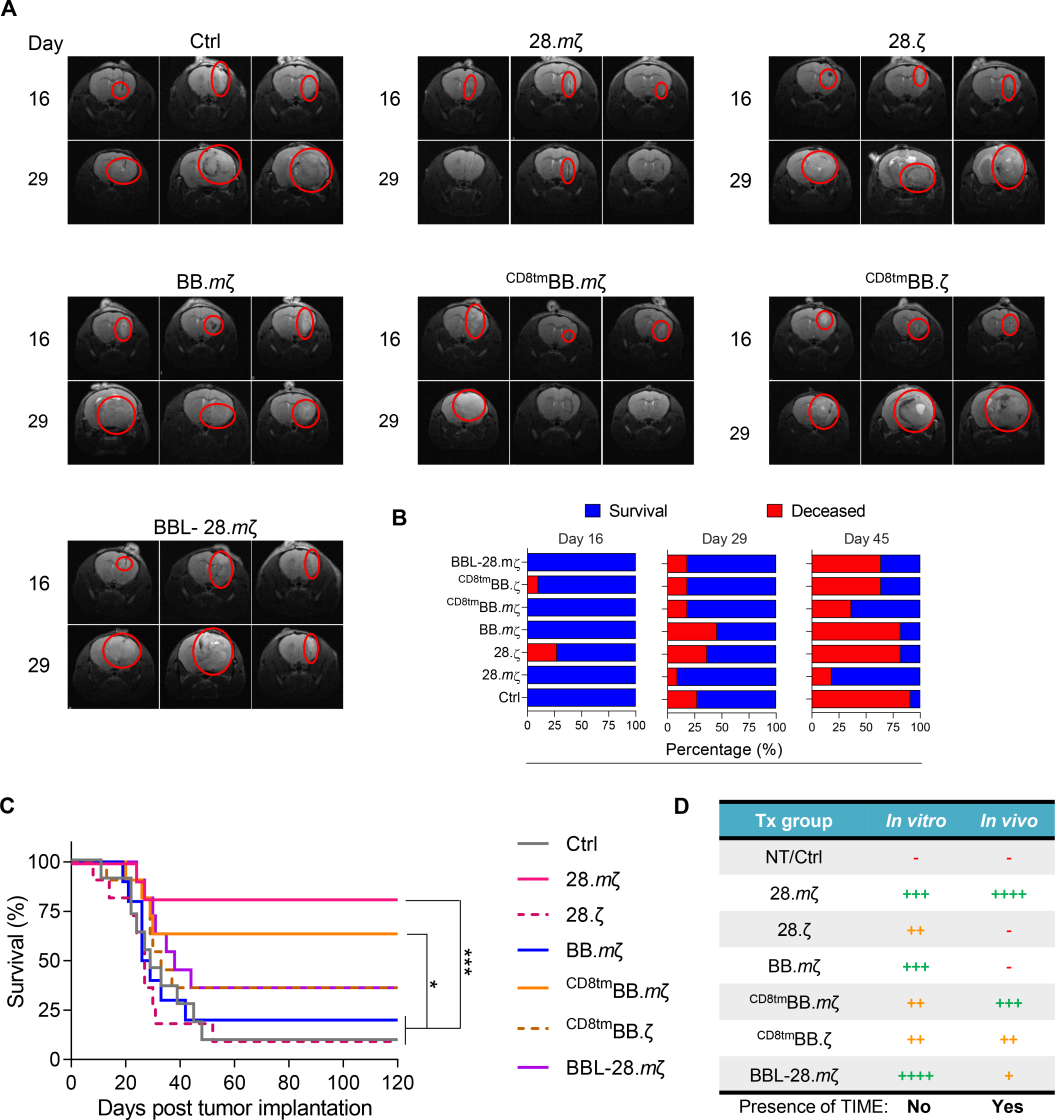
CAR structural design significantly impacts anti-glioma efficacy of mB7-H3 CAR T-cells in the GL261 immunocompetent model. Albino C57BL/6 mice were transplanted with 1 × 10^5^ GL261 cells orthotopically, followed 7 days later by intratumoral injection of 3 × 10^6^ mB7-H3-CAR T-cells transduced with different constructs and adjusted to 40% CAR expression. **A,** Axial brain MRI images from 3 representative mice per treatment group at days 16 and 29 after tumor implantation. **B,** Summary plots showing percentage of survival and deceased mice within each treatment group at days 16, 29, and 45 after tumor implantation. **C,** Kaplan–Meier survival curve (*n* = 11, log-rank Mantel–Cox test with Bonferroni correction for multiple comparisons, *, *P* < 0.05; ***, *P* < 0.001). Experiments were repeated twice with CAR T-cells generated from 2 different T-cell donors. **D,** Summary table for performance of different mB7-H3 CAR designs from *in vitro* and *in vivo* data [(−) means no response, increasing number of (+) signs mean better response].

Next, we evaluated whether optimizing CAR structure can enhance anti-glioma efficacy of mB7-H3 CAR T-cells *in vivo*. To avoid immunogenicity effects of luciferase and fluorescent proteins (28, 29), we used unmodified GL261 tumor cells. We used MRI to monitor tumor implantation and progression. On day 0, GL261 cells were implanted into the brains of C57BL/6 mice, followed by intratumoral injection of 3 × 10^6^ CAR T-cells on day 7. Treatment groups included Ctrl CAR and different mB7-H3 CARs depicted in Fig. 1A. *In vivo*, 28.*m*ζ CAR T-cells demonstrated the best overall control of tumor burden, which translated into enhanced survival (Fig. 3A–C, 28.*m*ζ vs^CD8tm^BB.*m*ζ *P* > 0.05; 28.*m*ζ vs. all other constructs *P* < 0.001). Interestingly, while ^CD8tm^BB.*m*ζ CAR T-cells did not perform well *in vitro*, they had comparable efficacy to the 28.*m*ζ CAR T-cells *in vivo* (Fig. 3A–C). Strikingly, BBL-28.*m*ζ CAR T-cells showed suboptimal anti-glioma responses *in vivo* (Fig. 3C, BBL-28.*m*ζ vs. 28.*m*ζ, ^CD8tm^BB.*m*ζ, or Ctrl *P* < 0.05; BBL-28.*m*ζ vs. all other constructs *P* > 0.05). A summary of our *in vitro* and *in vivo* findings is presented in Fig. 3D. Collectively, our data suggest that highly artificial *in vitro* system cannot accurately predict *in vivo* response in immunocompetent animal models.

### TIME Composition During CAR T-cell Treatment Depends on CAR Design

**FIGURE 4:**
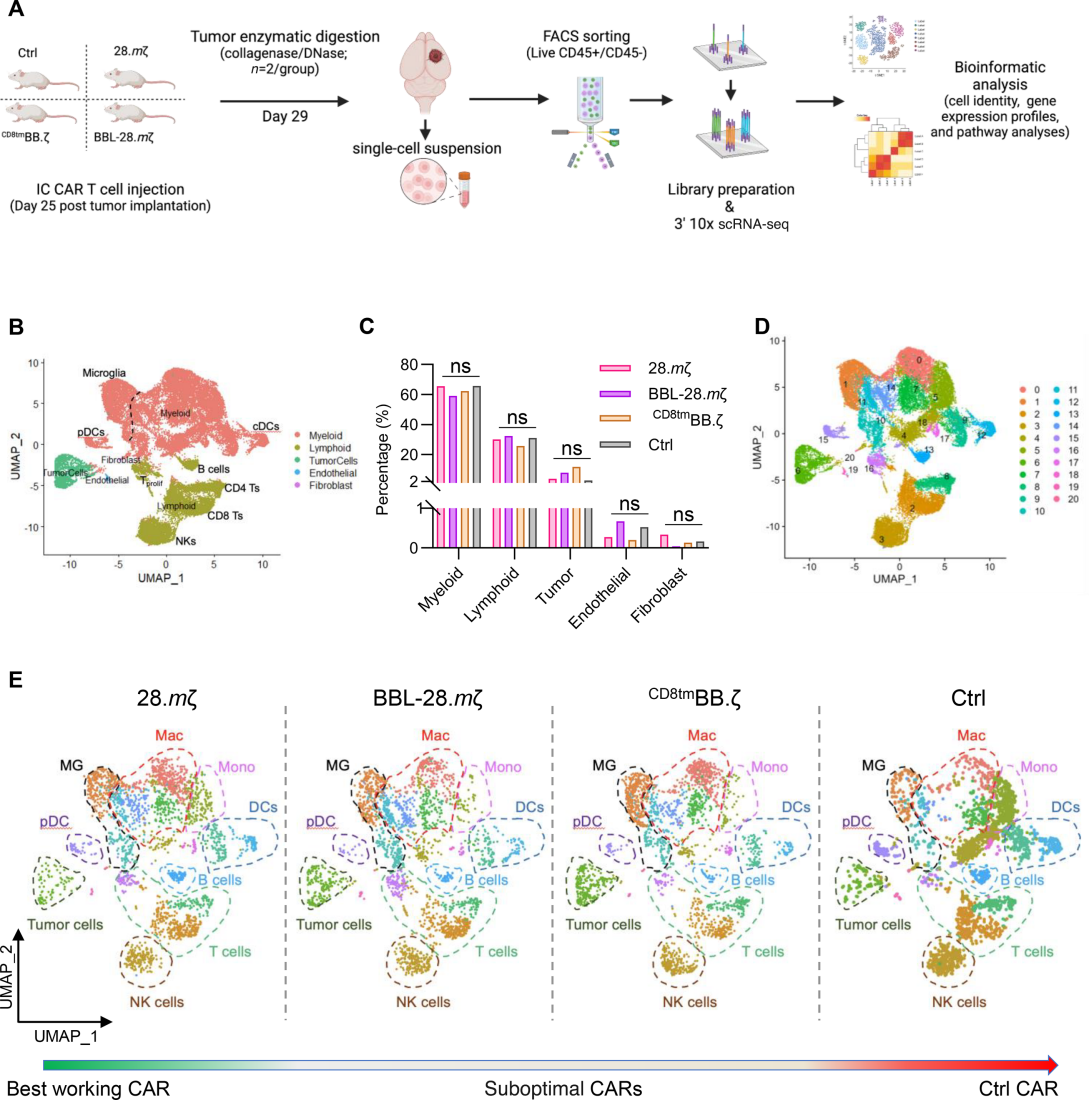
TIME heterogeneity after CAR T-cell treatment. **A,** Experimental scheme. Albino C57BL/6 mice were transplanted with 1 × 10^5^ GL261 cells orthotopically, followed 25 days later by intratumoral injection of 3 × 10^6^ mB7-H3-CAR T-cells (28.*m*ζ, BBL-28.*m*ζ,^CD8tm^BB.ζ, or Ctrl). Tumors were collected at 4 days after treatment and processed for scRNA-seq. Scheme created with BioRender.com. **B,** UMAP with major cell subsets in all tumor samples. **C,** Bar graph showing the percentage of each major cell type per treatment group. **D,** UMAP dimensionality reduction of single-cell data from all tumors clustered into 21 Seurat clusters annotated by number. **E,** UMAP visualization of the 21 Seurat clusters by treatment group from the best to the worst functioning CARs. Mac, macrophages; Mono, monocytes; MG, microglia; DC, dendritic cells.

We next wanted to assess whether the discrepancy that we observed between *in vitro* and *in vivo* responses was due to the glioma TIME. We performed scRNA-seq analyses. Tumors were collected at 4 days after treatment, processed, sorted for CD45^+^ and CD45^−^ fractions, and mixed at a ratio of 70% to 30%, respectively, for scRNA-seq (Fig. 4; Supplementary Fig. S6). We selected four CAR T-cell treatment groups that showed the best (28.*m*ζ, *n* = 2), suboptimal (BBL-28.*m*ζ, *n* = 1 and^CD8tm^BB.*m*ζ, *n* = 2) and no (Ctrl, *n* = 2) antitumor response (Fig. 4A; Supplementary Fig. S6A). Bioinformatic analyses revealed five major cell types based on hallmark gene expression: myeloid (*Cd11b*; 12 clusters), lymphoid (*Cd33/Ncr1/B220/Cd19*; five clusters), tumor (*Olig2/Cd276/Col11a1*; one cluster), endothelial (*Enpp2*; one cluster), and fibroblast (*plp1/Ptgds*; one cluster) cells (Fig. 4B). The myeloid compartment constituted the largest proportion of all clusters, with no differences in frequency among treatment groups (Fig. 4C). We next identified 21 transcriptionally unique cell clusters, including 16 distinct immune cell populations (Fig. 4D). Cluster identities were confirmed using ImmGen murine cell ID database, corroborating those identities determined by canonical genes for each cell type (Supplementary Fig. S7 and S8). We observed no apparent differences in cluster frequencies per treatment group (Fig. 4E; Supplementary Fig. S9). Thus, we next performed T-cell and macrophage subclustering analyses.

### T-cell Infiltrating Tumors That Failed CAR T-cell Therapy are Predominantly Exhausted

To analyze endogenous T-cell responses after CAR T-cell treatment, we further reclustered the CD4 and CD8 lymphoid compartment (clusters 2, 8, and 16 in Fig. 4D). T cells diversified into 11 subclusters with unique transcriptional profiles (Supplementary Fig. S10). First, tumors treated with Ctrl CAR had the largest proportion of naïve-like (C1: *Tcf7/Sell/Lef1*) and quiescent (C4: *S1pr1/Klf2/Slfn1*) T cells (Supplementary Fig. S10B–S10D). Second, BBL-28.*m*ζ and ^CD8tm^BB.ζ CAR-treated tumors were enriched in effector memory T cells, C0 and C6 (Supplementary Fig. S10B–S10D). Subclusters C0 and C6 expressed genes involved in immune effector processes like T-cell activation (*Cd28/C1qa/Lat*), cytokine signaling (*Zap70/Ifitm1/Ifngr1*), and cytotoxicity (*Gzma/Gzmb/Prf1*). However, these T cells also expressed multiple inhibitory receptors (*Pdcd1*/*Ctla4*/*Lag3*/*Havcr2*), *Eomes*, and *Tox.* Third, tumors treated with ^CD8tm^BB.ζ-CAR had larger proportions of regulatory (C2: *Foxp3/Tnfrsf9*) and effector (C7: *Ifng/Ccl1/Ccl4*) CD4 clusters. These CD4 clusters upregulated genes associated with antitumor immunity and chemotaxis (*Lat/Prf1/Zap70/Ccl3*) while simultaneously expressing genes associated with inhibitory T-cell responses (*Lag3/Havcr2/Tox/Ctla4*; Supplementary Fig. S10C and S10D). Collectively, endogenous T-cell responses in BBL-28.*m*ζ and ^CD8tm^BB.ζ groups were associated with a hyperactivated T-cell phenotype along with a concurrent expression of inhibitory/exhaustion signatures when compared with 28.*m*ζ-CAR group (Supplementary Fig. S11).

Using computational approaches, we next detected CAR antigen recognition domain (single-chain variable fragment) expression in macrophage, dendritic cell, and T-cell clusters (Supplementary Fig. S12A). Results suggest potential engulfing and uptake of CAR T-cells by antigen presenting cells. Focusing on T-cell subclusters, we found that CAR-positive cells within subclusters 3, 5, and 8 were mostly from tumors treated with 28.*m*ζ-CAR (Supplementary Fig. S12B). Subcluster 3 differentially upregulated genes associated with proliferation and cell-cycle progression (*Mki67/ Hist1h1d/Cenpa*) while subcluster 5 and 8 upregulated genes responsible for memory and cytolytic T-cell activity (C5: *Ly6c2*; C8: *Gzmc/Gzmf/Ccl1*; Supplementary Fig. S12C–S12E). Notably, ^CD8tm^BB.ζ-CAR was only detected at low frequencies in subclusters C3 and C8 (Supplementary Fig. S12B). Together, these data suggest that CAR T-cells posttreatment are highly proliferative, activated, and cytolytic.

### Not all Macrophage Responses After CAR T-cell Treatment are Suppressive

**FIGURE 5:**
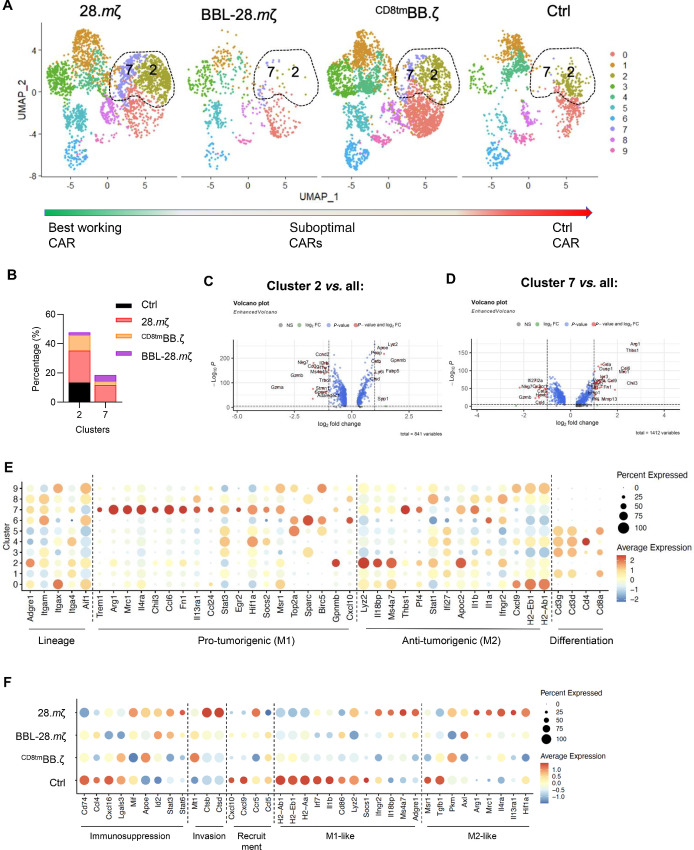
Successful responses with 28.*m*ζ CAR T-cells are associated with balanced proinflammatory and anti-inflammatory myeloid cell responses. Seurat clusters 0, 7, 10, 14 were reclustered into 10 Mac/MG subclusters to further define the diversity of myeloid responses after CAR T-cell treatment. **A,** UMAP plots of the Mac/MG subclusters visualized by treatment group. **B,** Summary plot of Mac/MG subcluster 2 and 7 frequencies per treatment. Volcano plots showing differentially upregulated and downregulated genes in macrophage subclusters C2 in **C**, C7 in **D** as compared with all other myeloid subclusters. **E,** Dot plot depicting expression of myeloid lineage markers, genes associated with protumorigenic responses, antitumorigenic, and differentiation genes. Dot size represents the percentage of cells expressing each gene and dot color represents mean expression level with a gradient of lowest expression in blue to highest expression in red. **F,** Dot plot depicting differentially expressed genes associated with immunosuppression, invasion, recruitment, M1-like and M2-like macrophage responses per treatment group.

Because macrophages are known to play an immunosuppressive role in the brain TIME (30), we next sought to further characterize the macrophage and macrophage-related compartments in our dataset. Cell-cell communication analysis [CellChat (27)] using our scRNA-seq dataset showed that macrophage/microglia (Mac/MG) clusters had the largest number and strength of interactions, suggesting their putative role in shaping the TIME after CAR T-cell treatment (Supplementary Fig. S13). We reclustered macrophage (Mac) and Mac/MG populations (C0, 7, 10, and 14 in Fig. 4D) resulting in 10 subclusters (Fig. 5A; Supplementary Fig. S14). While macrophage responses were heterogeneous, we observed that subclusters 2 and 7 were enriched in the 28.*m*ζ-CAR treatment group (Fig. 5B; C2: 21.77% in 28.*m*ζ, 1.73% in BBL-28.*m*ζ, 10.72 in ^CD8tm^BB.ζ and 13.42 in Ctrl; C7: 11.26% in 28.*m*ζ, 4.41% in BBL-28.*m*ζ, 2.59% in^CD8tm^BB.ζ, and 0.31% in Ctrl). Both subclusters expressed an array of anti- and proinflammatory genes simultaneously and were thus labeled as M1/M2 transitioning macrophages (Supplementary Fig. S14B and S14C). Subcluster 2 upregulated genes associated with inflammation (*Lyz2/Apoc2/Ifngr2/Irf7*) and immune regulation (*Arg1/Ccl8/Spp1/Gpnmb*; Fig. 5C and E). Subcluster 7 upregulated immunosuppressive (*Arg1/Mrc1/Chil3/Fn1*) and proinflammatory (*Cxcl2/Thbs1/Pf4/Il1b*) genes (Fig. 5D and E). Consequently, GSEA showed upregulation of hallmark pathways associated with inflammation (C2: complement, coagulation, reactive oxygen species; C7: inflammatory response, TNF signaling, glycolysis) along with downregulation of M2-macrophage pathways (C2 and C7: E2F targets, Myc targets, IFNalpha response) when compared with other subclusters (Supplementary Fig. S15A and S15B). Moreover, comparing macrophage responses in 28.*m*ζ-CAR group showed balanced activation of hallmark pathways associated with inflammatory immune responses compared with other groups (Supplementary Fig. S15C).

Finally, we noticed an interesting inverse pattern between 28.*m*ζ and Ctrl CAR treatments when looking at macrophage gene expression (Fig. 5F). Specifically, genes associated with immunosuppression (*Apoe/Id2/Stat3*), invasion (Mt1/*Ctsb/Ctsd)* and M2-like phenotype (Arg1/*Mrc1/Il4ra*) were downregulated in Ctrl CAR treatment group compared with 28.*m*ζ-CAR treatment. Conversely, genes associated with M1-like responses (*H2-Ab1/H2-Eb1/H2-Aa*) were downregulated in 28.*m*ζ-CAR compared with Ctrl CAR treatment. Together, our data show distinctive macrophage responses after CAR T-cell treatment that correlate with successful antitumor responses that are unique to TIME remodeling by CARs with specific costimulatory domains only.

### Nonresponding Mice Show Dysfunctional and Suppressive Immune Cells at Endpoint

**FIGURE 6:**
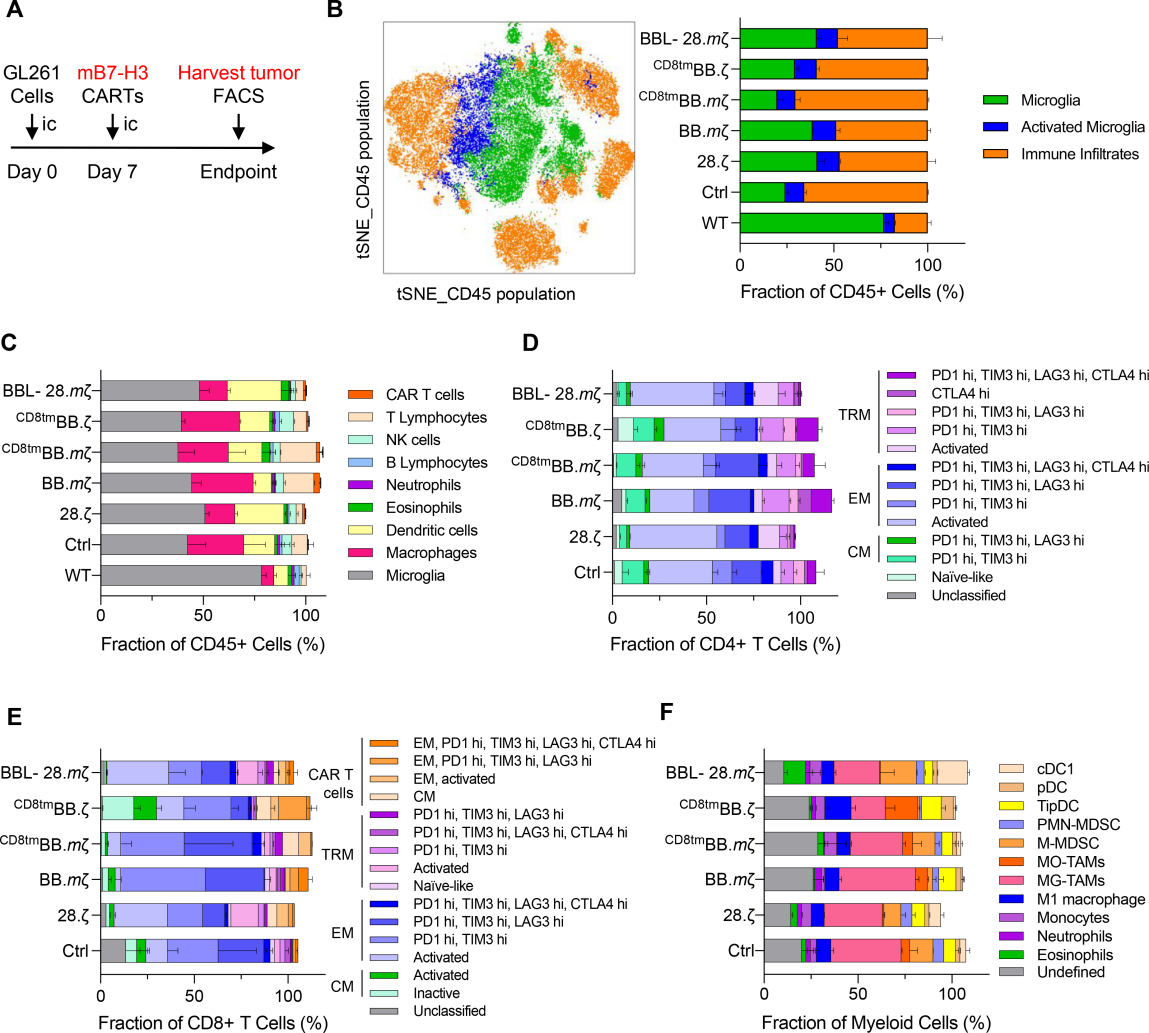
Comprehensive flow cytometry analysis of the TIME from tumors at endpoint. Unsupervised clustering analysis of flow data from tumors at endpoint was done using algorithmic analysis with FlowJo software for phenotyping heterogenous populations based on multi-parameter marker expression. **A,** Experimental scheme. **B,** Representative cluster plot showing diversity of CD45^+^ cells from tumors with bar graph depicting quantitative analysis in each treatment group (Microglia CD45^low^; Activated microglia CD45^low^, F4/80^low^, MHCII^high^, CD11b^low^; Immune cells CD45^high^). **C,** Graphical representation of the frequencies of major immune cell subsets per treatment group as defined by canonical cell surface markers. **D** and **E,** Quantitative plot of the CD4 and CD8 cell subsets after supervised subclustering analysis of lymphoid populations. **F,** Quantitative plot of myeloid cell populations per treatment group after supervised subclustering analysis. (cDC, conventional dendritic cells; pDC, plasmacytoid dendritic cells; TipDCs, TNF/iNOS-producing dendritic cells; PMN-MDSC, polymorphonuclear myeloid-derived suppressor cells; M-MDSC, monocytic myeloid-derived suppressor cells; MO-TAMs, monocyte-derived tumor associated macrophages; MG-TAMs, microglia-derived tumor associated macrophages).

To further understand changes in the TIME that contribute to limited therapeutic efficacy of mB7-H3 CAR T-cells, we investigated the immune composition of tumors at endpoint. Tumor samples were collected from nonresponding mice at the time of sacrifice and used for high-dimensional flow cytometry analyses (Fig. 6A). Samples included normal brains wild-type (WT) and tumors treated with different mB7-H3 CARs. Because 28.*m*ζ-CAR–treated tumors were completely eradicated, this group was not part of endpoint analyses. We observed an overall increase in CD45^+^ immune cells in CAR-treated tumors compared with normal brains (Fig. 6B). Specifically, there was an overall increase in macrophage, dendritic cell, B-cell, natural killer cell, and T-cell infiltrates within the TIME (Fig. 6C).

To identify distinct lymphoid populations, we stained tumor samples using a panel of comprehensive T-cell immunophenotyping markers and applied a multi-dimensional data-driven clustering algorithm to define unique populations. Consistent with scRNA-seq data from early timepoint, we found that mice treated with 41BB-CARs had higher proportions of T cells at endpoint compared with mice treated with CD28-CARs (Fig. 6C). Specifically, data show a larger proportion of central memory CD4^+^ T cells in tumors treated with 41BB-CARs (Fig. 6D). In addition, larger percentages of CD8^+^ effector T cells infiltrated tumors treated with BBL-28.*m*ζ CAR (Fig. 6D). Yet, most T-cell subsets (including CAR T-cells) expressed multiple exhaustion markers indicating that persisting T cells at endpoint are dysfunctional (Fig. 6D and E).

To delineate specific phenotypes of infiltrating cells, we did multi-dimensional subclustering of myeloid populations. We found that the majority of macrophages in nonresponding mice were microglia-derived (MG-TAM, *CD49d^lo^*^w^; Fig. 6F). Similar to scRNA-seq data from early timepoints, MG-TAMs in BBL-28.*m*ζ and ^CD8tm^BB.ζ groups intracellularly expressed M2-like suppressive markers (Arg1/Il10/Ahr; Supplementary Fig. S16A–S16C). In addition, M1/M2 transitioning macrophages concurrently expressing proinflammatory and anti-inflammatory markers (Arg1/Il10/Ahr/Il17/Ifng/Tnfa/IL6) were also present at endpoint (Supplementary Fig. S16C). Thus, our flow cytometry data from tumors at endpoint reiterate the potential role of endogenous myeloid and T-cell populations in dictating successful responses to CARs with distinct structural domains.

### Global Macrophage Depletion Abrogates CAR T-cell Antitumor Efficacy

**FIGURE 7:**
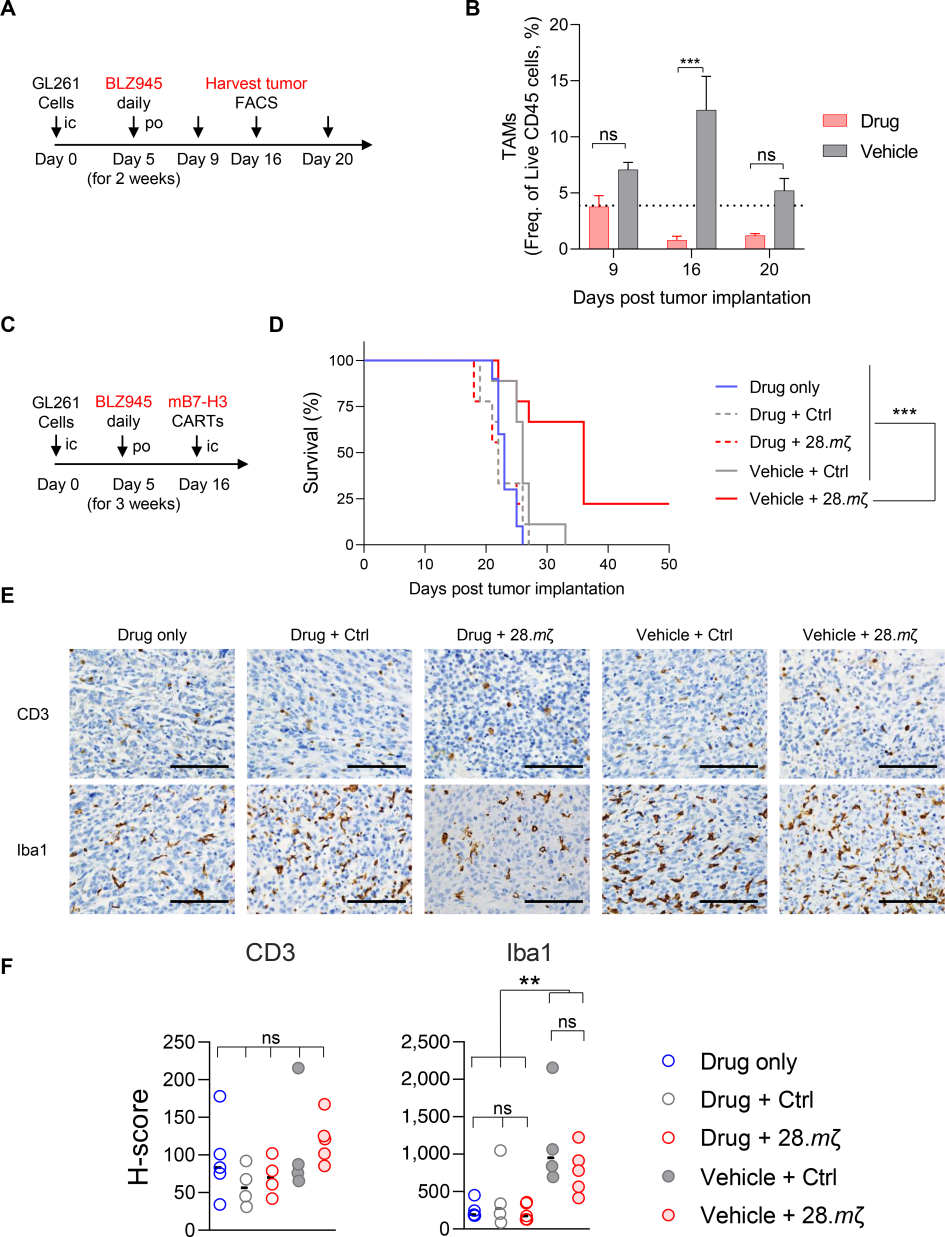
Global Mac/MG depletion abrogates effective CAR T-cell responses. GL261 glioma-bearing mice were treated with BLZ945 at 200 mg/kg starting 5 days after tumor implantation. **A,** Experimental scheme of BLZ945 macrophage depletion kinetics experiment. Daily drug dosing via oral gavage was for 2 weeks and tumors were harvested for FACS analysis at days 9, 16, and 20 after tumor implantation. **B,** Summary plot showing frequency of TAMs infiltrating tumors as percentage of live CD45^+^ immune cells. **C,** Experimental scheme for combination study. Glioma-bearing mice were treated with BLZ945 at 200 mg/kg starting 5 days after tumor implantation and continued daily for 3 weeks. B7-H3 CAR T-cells with Ctrl or 28.*m*ζ constructs were then injected intratumorally at day 16. **D,** Kaplan–Meier survival curve (*n* = 11, log-rank Mantel–Cox test with Bonferroni correction for multiple comparisons, ***, *P* < 0.001). **E,** Representative images from immunostaining for T-cell and macrophage markers from tumors at endpoint showing CD3 and Iba1 staining in brain samples from each treatment group at 40x magnification (scale bar = 100 µm). **F,** H-scores depicting quantitative analysis of CD3 and Iba1 staining in brain tumor samples at endpoint from E as evaluated by blinded pathologist.

Because macrophages were particularly prominent in our TIME studies, and as there is currently no method to target and deplete specific macrophage clusters, such as subclusters 2 and 7, we opted for a comprehensive macrophage depletion approach by employing the CSF1R inhibitor, BLZ945. This drug has been shown to effectively deplete spinal cord and brain macrophages as well as microglia in brain tumor models (31). Importantly, we found that BLZ945 does not affect CAR T-cell functions *in vitro* (Supplementary Fig. S17 and S18). *In vivo*, daily treatment with 200 mg/kg of BLZ945 in glioma-bearing mice effectively depleted all macrophages following 11 days of consecutive dosing (Fig. 7A and B, *P* = 0.0005). Therefore, we evaluated whether 11-day pretreatment with BLZ945 would affect anti-glioma efficacy of 28.*m*ζ CAR T-cells. Interestingly, macrophage depletion had a negative impact on tumor control and survival in mice receiving BLZ945 in combination with mB7-H3 CAR T-cells (Fig. 7C and D; Supplementary Fig. S19A). Immunostaining of tumors from endpoint revealed that macrophages (Iba1, F4/80) continue to be depleted in groups treated with BLZ945, while T-cell (CD3) infiltration was not impacted (Fig. 7E and F; Supplementary Fig. S19B and S19C).

To confirm the detrimental effects of BLZ945 on CAR T-cell efficacy were not related to larger tumor volumes due to the delayed CAR T-cell treatment timeline, we performed the same combination therapy but started BLZ945 treatment at 4 days prior to tumor implantation, and injected CAR T-cells intratumorally at 7 days after implantation (Supplementary Fig. S19D). Similarly, global macrophage depletion continued to have a detrimental effect on CAR T-cell therapy in glioma-bearing mice (Supplementary Fig. S19E). Together, these data expand upon our TIME analyses to confirm that specific macrophage subsets are essential for CAR antitumor responses.

## Discussion

The potential of T-cell immunotherapies to induce complete responses in brain tumors depends on their ability to elicit robust and sustained effector functions within the hostile TIME (32). In addition, durable immune responses require differentiation of infused CAR T-cells into effector and memory phenotypes that allows them to persist in the highly suppressive microenvironment (33). Here, we show that optimizing CAR design based on *in vitro* profiling is not sufficient to induce superior responses in the presence of the TIME. *In vitro* experiments demonstrate that 28.*m*ζ CAR T-cells have the best overall cytotoxicity and that incorporating 41BBL signaling in 28.*m*ζ CAR T-cells enhances their expansion and persistence. To our surprise, BBL-28.*m*ζ CAR T-cells did not have any superior survival advantage regardless of its phenomenal performance *in vitro*. Our scRNA-seq analyses of tumors treated with CAR T-cells containing different functional domains revealed unique changes in the TIME composition. We found that two transcriptionally distinct macrophage clusters (subclusters 2 and 7) were enriched in tumors treated with the best functioning CAR (28.*m*ζ), suggesting that the presence of specific macrophage populations mediates successful CAR T-cell responses. Indeed, tumors depleted of macrophages and treated with 28.*m*ζ-CARs failed to respond to therapy.

It is well established that different CAR functional domains have strength and limitations when it comes to CAR T-cell effector functions. For example, CD28 domain provides faster and more profound activation of T-cell effector mechanisms due to enhanced changes in protein phosphorylation events compared with 41BB domain (34). Moreover, CD3ζ can result in tonic CAR activation and early exhaustion (35). Thus, calibrating CAR signaling by mutating one or two ITAMs can balance baseline CAR activation (36, 37). Finally, incorporating 41BB signaling into CD28-CARs using *in trans* design enhances therapeutic efficacy and persistence of CAR T-cells in leukemia and solid tumor models (38). Our data reported here add new knowledge to these existing findings. Specifically, we show that besides differential CAR T-cell signaling, the choice of CAR functional domains uniquely remodels the TIME and results in differential antitumor responses.

Macrophages heavily infiltrate gliomas and are associated with tumorigenesis and treatment resistance (39). However, macrophage populations are highly heterogenous and perform diverse functions ranging from enhancing tumor progression (enhanced tumor proliferation and invasion) to inducing immune evasion (restricting lymphoid infiltrates and promoting regulatory T-cell phenotypes). Moreover, macrophages are a major source of type I and II IFNs which have direct antitumor effects and the potential to coordinate anti-inflammatory responses. Thus, macrophages are classified into classical (M1) and alternative (M2) phenotypes (40). Although macrophage polarization is a dynamic state, M1-macrophages are considered proinflammatory and express markers associated with immune responses (Tnfa, Ifng, MhcII); while M2-macrophages are suppressive with characterized expression of immune regulatory markers (Arg1, Il-10, Cd206). In our study, we found 10 transcriptionally distinct macrophage subclusters present in tumors after CAR T-cell treatment and none of them fit perfectly in the classical M1- or M2-macrophage classifications reiterating the dynamic functions of macrophages within the TIME. As such, we find it essential to incorporate high-throughput analyses (such as scRNA-seq and high-dimensional flow) into characterizing the brain TIME as it provides new insights into functional immune signatures. These findings may challenge traditional macrophage classification but will provide new insights into currently unknown macrophage functions within the brain TIME. It is important to replicate these studies for each unique tumor type to delineate macrophage interactions with adoptive immunotherapies in distinct tissues and etiologies.

In the past decade, an unprecedented number of genetic modifications have emerged significantly advancing T cell–based immunotherapies (41). Most of them are aimed at improving T-cell signaling strength (42, 43), persistence and expansion (44, 45), or sensitivity to antigen density (46). The standard approach for evaluating all new modifications is to first evaluate the improved functions *in vitro*, followed by *in vivo* testing in immunodeficient mouse models. Typically, *in vitro* findings translate into enhanced antitumor efficacy *in vivo*. However, only few studies verified new modifications in immunocompetent models (47–49). Our study was exclusively performed in an immunocompetent brain tumor model using fully syngeneic CAR constructs. In this system, we found that *in vivo* results did not mirror our *in vitro* findings. Previous studies, including one from our group, showed that 41BBL does indeed improve CAR T-cell functions when tested in xenograft models (3, 20). These findings urge us to ask the following questions: Will all new CAR designs be evaluated in immunocompetent animal models for findings relevant to clinical applications? Does the brain microenvironment shut down highly potent CAR T-cells to protect it from CAR-induced inflammation? Are we over-engineering our CARs by making them more potent?

In conclusion, our results show that 28.*m*ζ-CAR have superior efficacy in immunocompetent glioma models. Analysis of the TIME revealed that presence of immune cell hubs consisting of macrophages and endogenous T cells are associated with successful therapeutic responses to adoptive immunotherapies. Moreover, data from *in vivo* testing confirm that while helpful, *in vitro* results do not accurately predict how specific CAR domains function within the brain TIME. Finally, our study underscores the significance of investigating CAR T-cell functionality in models that have a functional immune system.

### Limitations of the Study

This study primarily focuses on CAR design and its impact on the TIME within one immunocompetent glioma model (GL261), presenting some noteworthy limitations. The GL261 model is widely accepted among researchers due to its consistent tumor growth, extensive characterization in the literature, exogenous expression of B7-H3, and its ability to faithfully recapitulate glioma histology, making it a good choice for our initial studies (50, 51). However, extending the validation of key findings to other glioma and brain tumor models is imperative for understanding the extent and the impact of the TIME on CAR T-cell function. Thus, future studies will evaluate these findings in other immunocompetent brain tumor models to establish broader applicability. Second, the study lacks mechanistic validation of the functions and interactions of C2 and C7 macrophage subclusters, leaving an important aspect unexplored. Despite these limitations, this research delivers timely and essential insights into CAR T-cell immunotherapy for brain tumors, with ongoing efforts dedicated to addressing these gaps in understanding.

## Supplementary Material

Supplementary Table S1 Supplementary Table S1

Supplementary Table S2 Supplementary Table S2

Supplementary Figure 1 Supplementary Figure S1 shows murine CAR T cell efficacy in vivo and Cd276 IHC post-treatment.

Supplementary Figure 2 Supplementary Figure S2 shows schematic of mB7-H3-CAR constructs.

Supplementary Figure 3 Supplementary Figure S3 shows expression of murine B7-H3 CARs in producer cells and T cells.

Supplementary Figure 4 Supplementary Figure S4 shows phenotype and cytotoxicity of murine B7-H3 CAR T cells.

Supplementary Figure 5 Supplementary Figure S5 shows murine CAR T cell expansion in media and after exposure to B7-H3-negative tumor cells.

Supplementary Figure 6 Supplementary Figure S6 shows experimental pipeline for single cell RNAseq experiment.

Supplementary Figure 7 Supplementary Figure S7 shows initial analysis of scRNAseq data.

Supplementary Figure 8 Supplementary Figure S8 provides cluster identities.

Supplementary Figure 9 Supplementary Figure S9 shows cluster frequency per treatment group for each cluster.

Supplementary Figure 10 Supplementary Figure S10 shows diversity of T cell clusters per treatment group.

Supplementary Figure 11 Supplementary Figure S11 shows dot plots depicting differentially expressed T cell genes in T cell clusters per treatment group.

Supplementary Figure 12 Supplementary Figure S12 shows CAR presence in each cluster and GSEA analysis in specific clusters.

Supplementary Figure 13 Supplementary Figure S13 shows cell-cell communication analysis within the TIME from our scRNAseq dataset.

Supplementary Figure 14 Supplementary Figure S14 shows macrophage populations post CAR T-cell treatment in GL261 glioma bearing mice.

Supplementary Figure 15 Supplementary Figure S15 shows GSEA of hallmark pathways in macrophage subclusters.

Supplementary Figure 16 Supplementary Figure S16 shows intracellular flow cytometry data from the endpoint tumors after CAR T cell treatment.

Supplementary Figure 17 Supplementary Figure S17 shows testing of BLZ945 effect on CAR T cell in vitro.

Supplementary Figure 18 Supplementary Figure S18 shows BLZ945 effect on CAR T cells in media and in co-culture with GL261-KO cells.

Supplementary Figure 19 Supplementary Figure S19 shows IHC for F4/80 and CD11c after BLZ945 and CAR T cell treatment, and OS post combination treatment.
