# Supplementary material for: CAR T-cell Design-dependent Remodeling of the Brain Tumor Immune Microenvironment Modulates Tumor-associated Macrophages and Anti-glioma Activity
Source: Cancer Res Commun. 2023 Dec 1;3(12):2430–46. doi: 10.1158/2767-9764.CRC-23-0424 (PMC10689147; doi:10.1158/2767-9764.CRC-23-0424)
Supplement: Supplementary Figure 1 — Supplementary Figure S1 shows murine CAR T cell efficacy in vivo and Cd276 IHC post-treatment. [file crc-23-0424-s03.pdf]

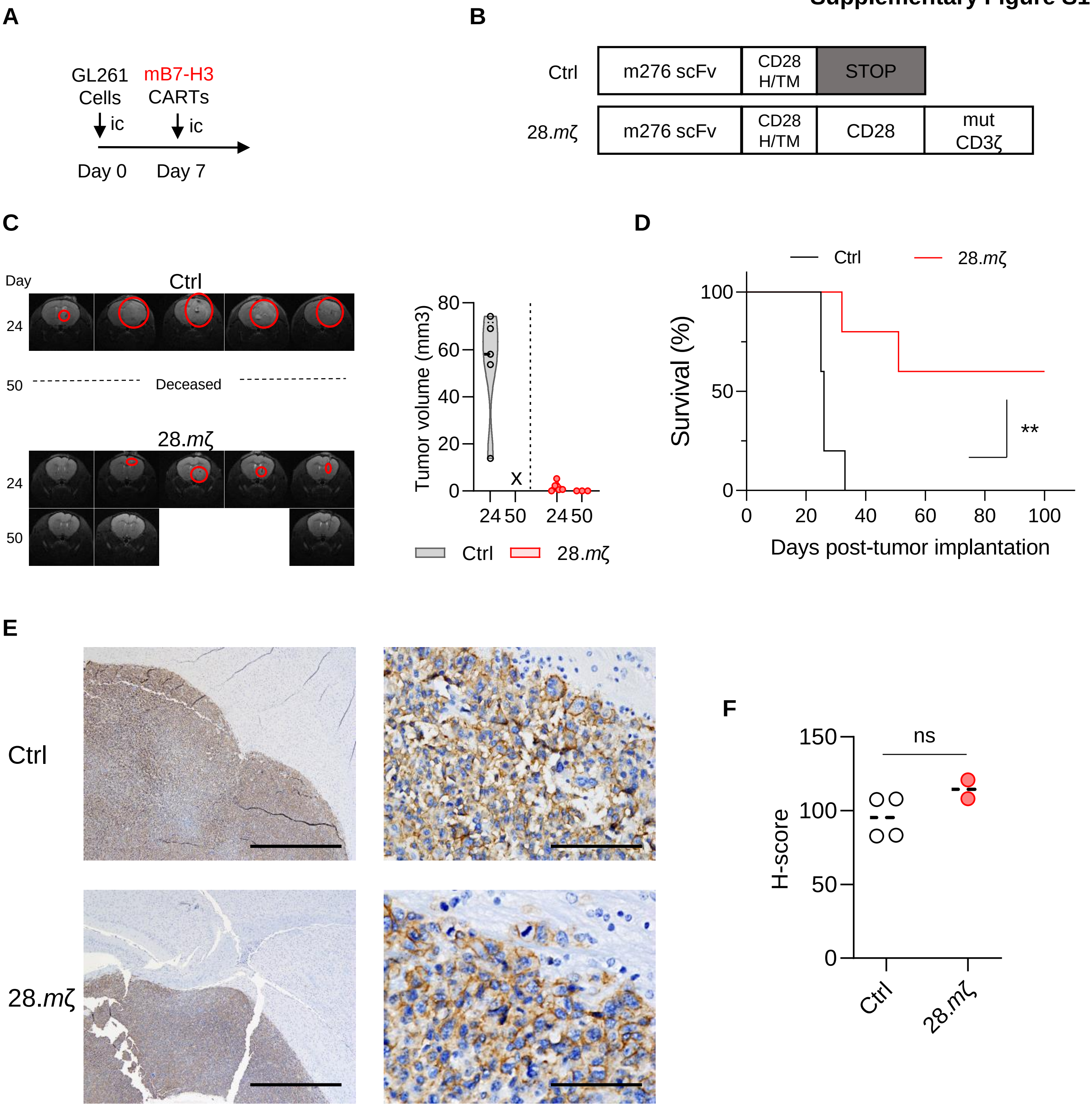

Supplementary Fig. S1: CD28-based B7-H3 CAR T-cells with mutated CD3ζ domain have modest anti-glioma efficacy in the immunocompetent GL261 model. Albino C57BL/6 mice were transplanted with 1×10^5 GL261 cells orthotopically, followed 7-days later by intra-tumoral injection of 3×10^6 mB7-H3 (28.*m*ζ) or control (Ctrl) CAR T-cells adjusted to 40% CAR expression. **(A)** Experimental design. **(B)** Scheme of mB7-H3 and Ctrl CAR constructs. **(C)** Axial brain MRI images with quantitative violin plots of tumor volume measurements from days 24 and 50 post-tumor implantation. **(D)** Kaplan-Meier survival curve (n = 5, log-rank Mantel-Cox test with Bonferroni's correction for multiple comparisons, **P<0.001). **(E)** Representative IHC images of brain sections stained for murine B7-H3 expression from Ctrl and 28.*m*ζ CAR-treated groups at 10x and 40x, respectively (scale bar = 400 μm and 100 μm , respectively). **(F)** H-scores for B7-H3 staining intensity from multiple mice at endpoint.
