# Supplementary material for: CAR T-cell Design-dependent Remodeling of the Brain Tumor Immune Microenvironment Modulates Tumor-associated Macrophages and Anti-glioma Activity
Source: Cancer Res Commun. 2023 Dec 1;3(12):2430–46. doi: 10.1158/2767-9764.CRC-23-0424 (PMC10689147; doi:10.1158/2767-9764.CRC-23-0424)
Supplement: Supplementary Figure 2 — Supplementary Figure S2 shows schematic of mB7-H3-CAR constructs. [file crc-23-0424-s04.pdf]

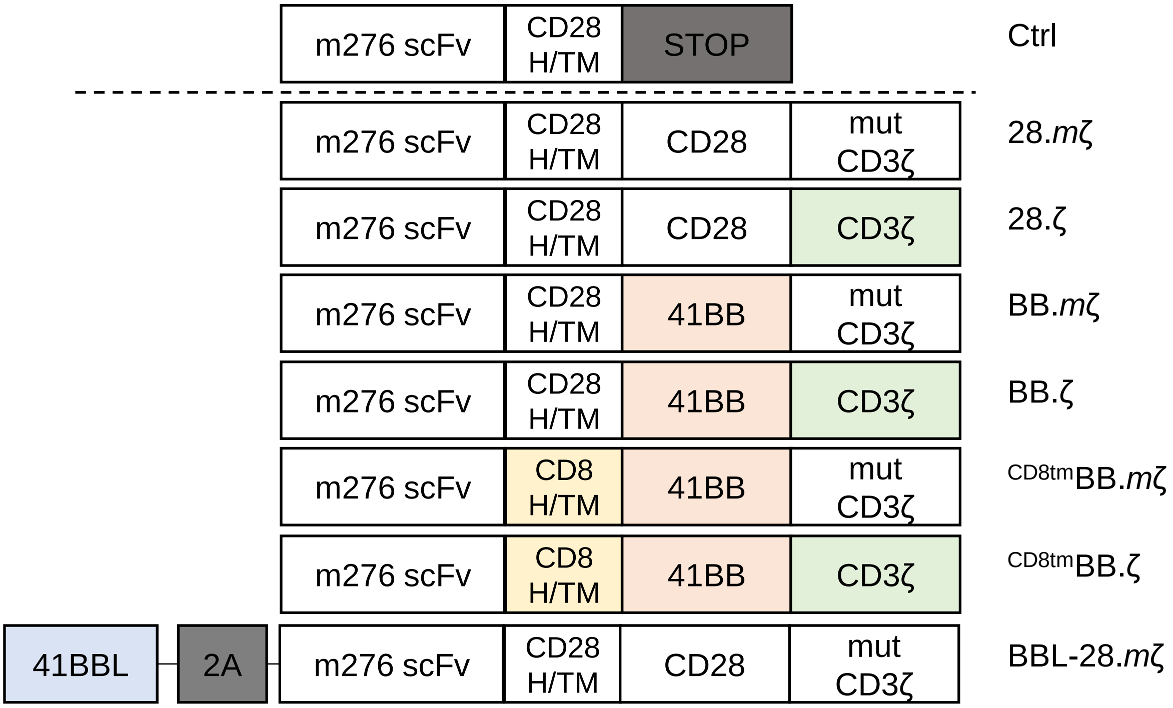

Supplementary Fig. S2: Generating a library of syngeneic B7-H3 CARs with different transmembrane, costimulatory, and activation domains. Scheme of mB7-H3-CAR constructs.
