# Supplementary material for: CAR T-cell Design-dependent Remodeling of the Brain Tumor Immune Microenvironment Modulates Tumor-associated Macrophages and Anti-glioma Activity
Source: Cancer Res Commun. 2023 Dec 1;3(12):2430–46. doi: 10.1158/2767-9764.CRC-23-0424 (PMC10689147; doi:10.1158/2767-9764.CRC-23-0424)
Supplement: Supplementary Figure 3 — Supplementary Figure S3 shows expression of murine B7-H3 CARs in producer cells and T cells. [file crc-23-0424-s05.pdf]

Supplementary Figure S3

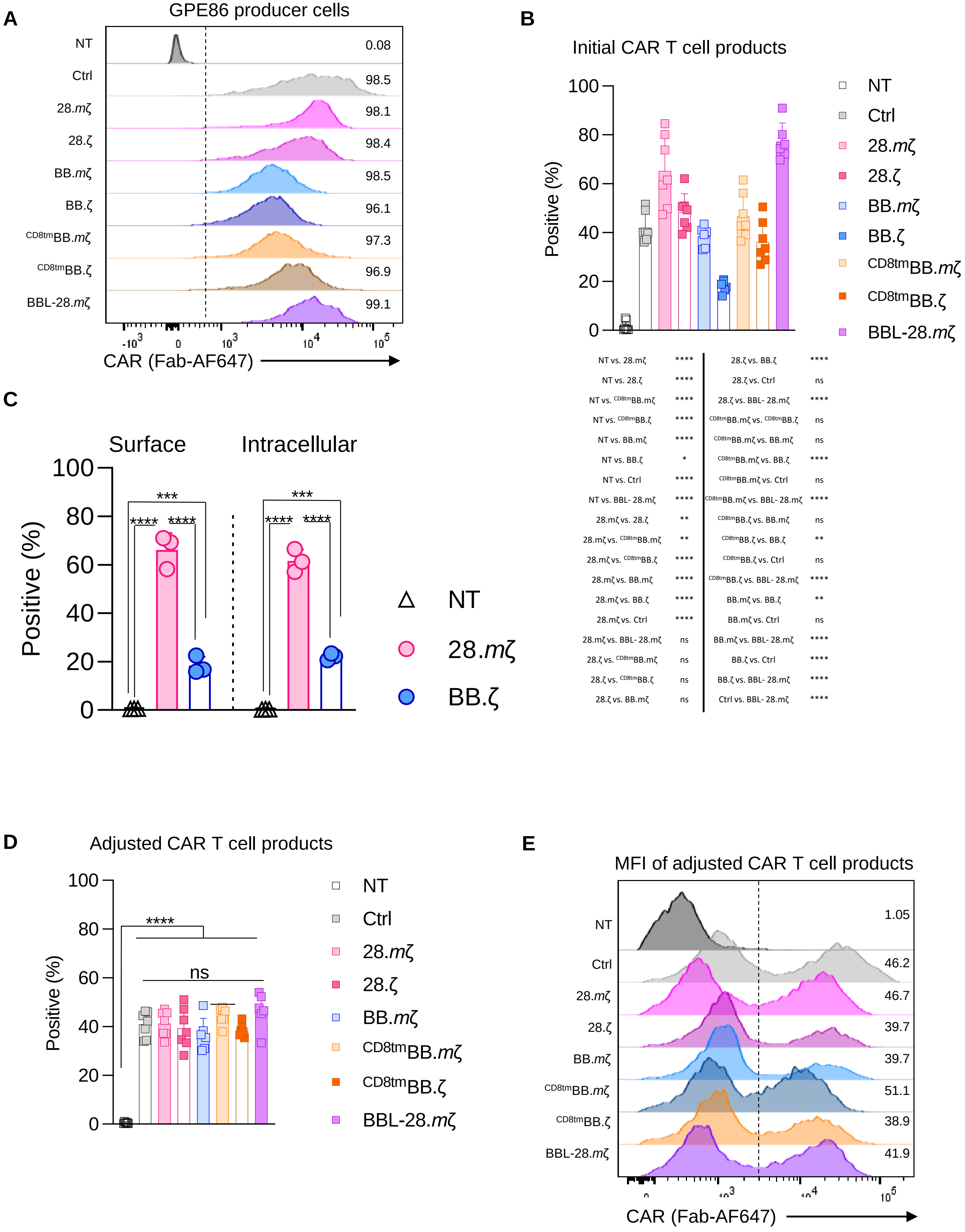

NT vs. 28.mζ	****	28.ζ vs. BB.ζ	****
NT vs. 28.ζ	****	28.ζ vs. Ctrl	ns
NT vs. CD8tmBB.mζ	****	28.ζ vs. BBL- 28.mζ	****
NT vs. CD8tmBB.ζ	****	CD8tmBB.mζ vs. CD8tmBB.ζ	ns
NT vs. BB.mζ	****	CD8tmBB.mζ vs. BB.mζ	ns
NT vs. BB.ζ	*	CD8tmBB.mζ vs. BB.ζ	****
NT vs. Ctrl	****	CD8tmBB.mζ vs. Ctrl	ns
NT vs. BBL- 28.mζ	****	CD8tmBB.mζ vs. BBL- 28.mζ	****
28.mζ vs. 28.ζ	**	CD8tmBB.ζ vs. BB.mζ	ns
28.mζ vs. CD8tmBB.mζ	**	CD8tmBB.ζ vs. BB.ζ	**
28.mζ vs. CD8tmBB.ζ	****	CD8tmBB.ζ vs. Ctrl	ns
28.mζ vs. BB.mζ	****	CD8tmBB.ζ vs. BBL- 28.mζ	****
28.mζ vs. BB.ζ	****	BB.mζ vs. BB.ζ	**
28.mζ vs. Ctrl	****	BB.mζ vs. Ctrl	ns
28.mζ vs. BBL- 28.mζ	ns	BB.mζ vs. BBL- 28.mζ	****
28.ζ vs. CD8tmBB.mζ	ns	BB.ζ vs. Ctrl	****
28.ζ vs. CD8tmBB.ζ	ns	BB.ζ vs. BBL- 28.mζ	****
28.ζ vs. BB.mζ	ns	Ctrl vs. BBL- 28.mζ	****

Supplementary Fig. S3: Design and expression of syngeneic B7-H3 CARs with different transmembrane, costimulatory, and activation domains. **(A)** Representative flow plots of GPE-86 producer cell lines expressing different CAR constructs post FACS sorting for the top positive cells. **(B)** Summary plot of %F(ab′)2-positive T-cells at 3-5 days post transduction (n = 7, mean ± SD, 2-way ANOVA with Tukey's test for multiple comparisons, table shows statistical comparisons between groups. * $p<0.05$; ** $p<0.01$; *** $p<0.001$; **** $p<0.0001$; ns, non-significant.). (C) Quantitative bar graph of %F(ab′)2-positive T cells from surface and intracellular staining of B7-H3 CAR T cells expressing 28.mζ and BB.ζ CARs. (D) Summary plot of %F(ab′)2-positive T-cells titrated to 40% CAR expression with NT T-cells (n = 7, mean ± SD, 1-way ANOVA with Tukey's test for multiple comparisons, $p<0.0001$). (E) Representative flow plots of %F(ab′)2-positive T cells titrated to an average of 40% CAR expression with NT T cells.
