# Supplementary material for: CAR T-cell Design-dependent Remodeling of the Brain Tumor Immune Microenvironment Modulates Tumor-associated Macrophages and Anti-glioma Activity
Source: Cancer Res Commun. 2023 Dec 1;3(12):2430–46. doi: 10.1158/2767-9764.CRC-23-0424 (PMC10689147; doi:10.1158/2767-9764.CRC-23-0424)
Supplement: Supplementary Figure 4 — Supplementary Figure S4 shows phenotype and cytotoxicity of murine B7-H3 CAR T cells. [file crc-23-0424-s06.pdf]

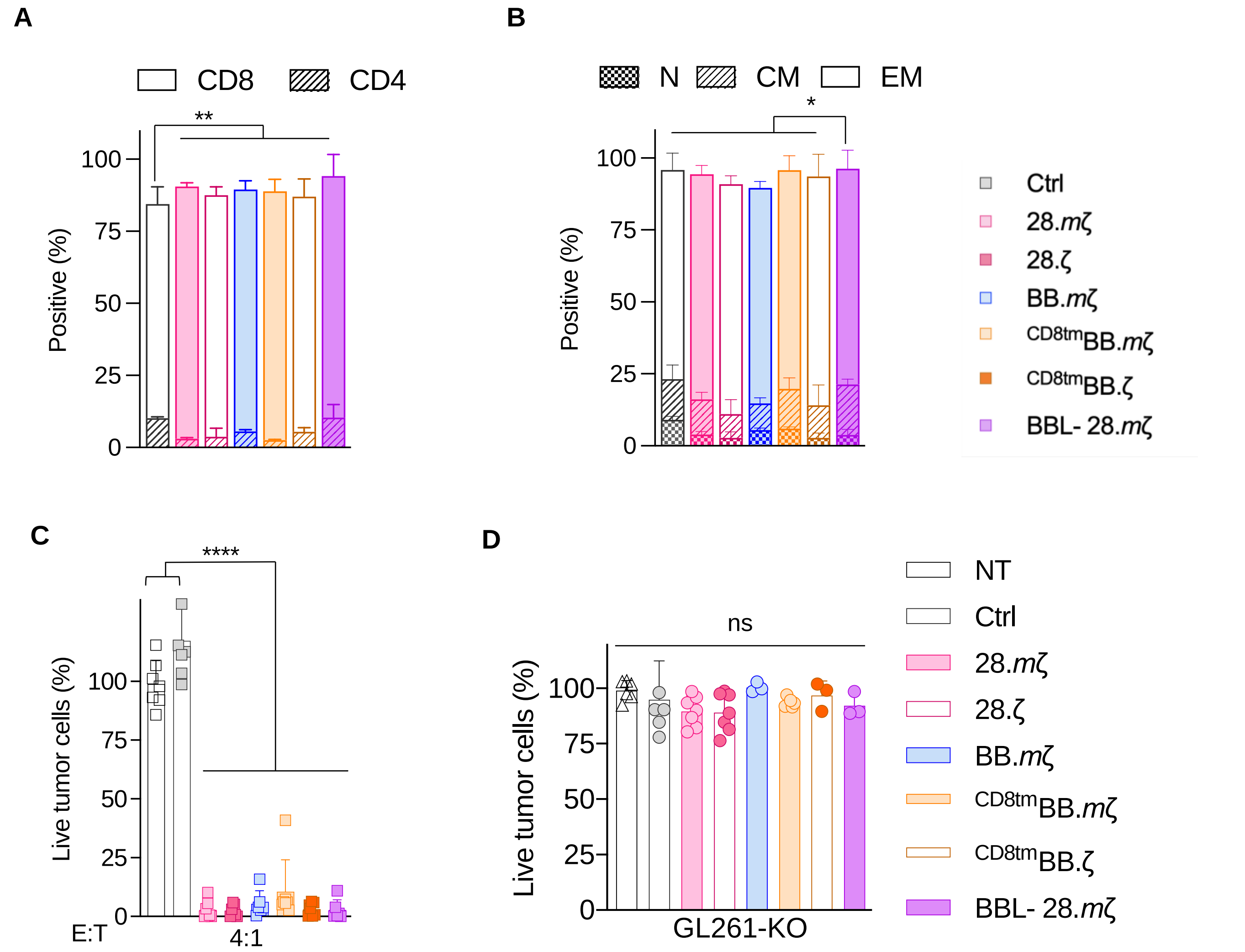

Supplementary Fig. S4: Functional characterization of mB7-H3-CAR T cells with different domains **(A)** Summary plot of CD4 and CD8 composition of mB7-H3-CAR products at day 5 post-transduction (n = 4, mean ± SD, 2-way ANOVA with Tukey's test for multiple comparisons). **(B)** Summary plot of memory phenotypes (Effector memory (EM): CD44+/CD62L−, central memory (CM): CD44+/CD62L+, naïve (N): CD44−/CD62L) of mB7-H3-CAR products (n = 4, mean ± SD, 2-way ANOVA with Tukey's test for multiple comparisons). **(C)** MTS cytotoxicity assay against GL261 tumor cells at an effector to target (E:T) ratio of 4:1 (n = 7, mean ± SD, 2-way ANOVA with Tukey's test for multiple comparisons). **(D)** MTS cytotoxicity assay against GL261 *B7h3* knockout (GL261-KO) tumor cells at an E:T ratio of 4:1 (n = 6, mean ± SD, 2-way ANOVA with Tukey's test for multiple comparisons.
