# Supplementary material for: CAR T-cell Design-dependent Remodeling of the Brain Tumor Immune Microenvironment Modulates Tumor-associated Macrophages and Anti-glioma Activity
Source: Cancer Res Commun. 2023 Dec 1;3(12):2430–46. doi: 10.1158/2767-9764.CRC-23-0424 (PMC10689147; doi:10.1158/2767-9764.CRC-23-0424)
Supplement: Supplementary Figure 5 — Supplementary Figure S5 shows murine CAR T cell expansion in media and after exposure to B7-H3-negative tumor cells. [file crc-23-0424-s07.pdf]

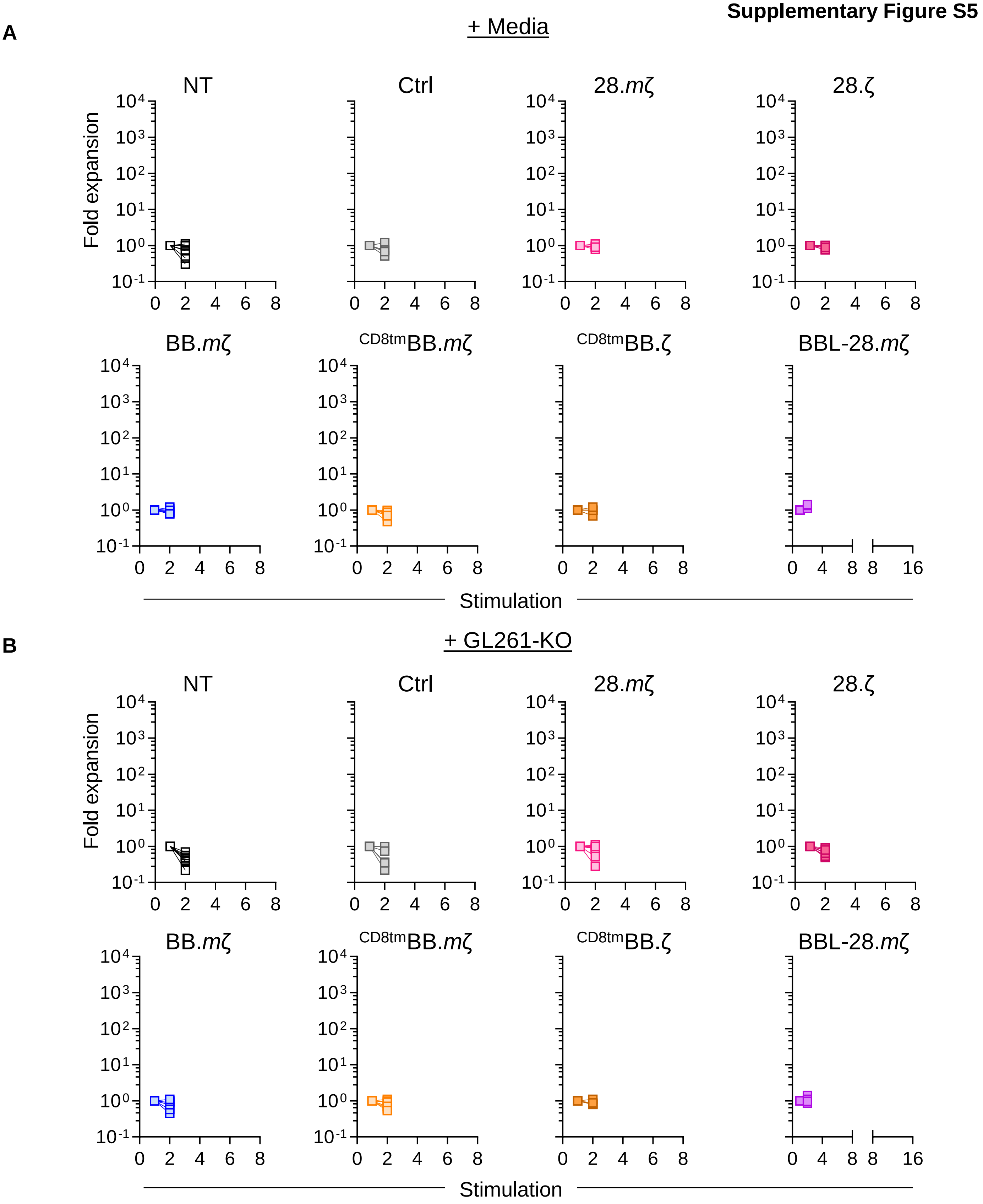

Supplementary Fig. S5: T-cells expressing different mB7-H3 different CAR constructs expand in an antigen-dependent manner. Transduced T-cells were cocultured with media only or GL261 *B7h3*-KO tumor cells at a 2:1 ratio with restimulation every 3-days against fresh tumor cells until they no longer killed and/or expanded. **(A)** Fold expansion of different T-cell donors in the absence of target cells (n = 5). **(B)** Expansion of different T-cell donors upon stimulation with antigen-negative GL261 *B7h3*-KO tumor cells (n = 7).
