# Supplementary material for: CAR T-cell Design-dependent Remodeling of the Brain Tumor Immune Microenvironment Modulates Tumor-associated Macrophages and Anti-glioma Activity
Source: Cancer Res Commun. 2023 Dec 1;3(12):2430–46. doi: 10.1158/2767-9764.CRC-23-0424 (PMC10689147; doi:10.1158/2767-9764.CRC-23-0424)
Supplement: Supplementary Figure 6 — Supplementary Figure S6 shows experimental pipeline for single cell RNAseq experiment. [file crc-23-0424-s08.pdf]

Supplementary Figure S6

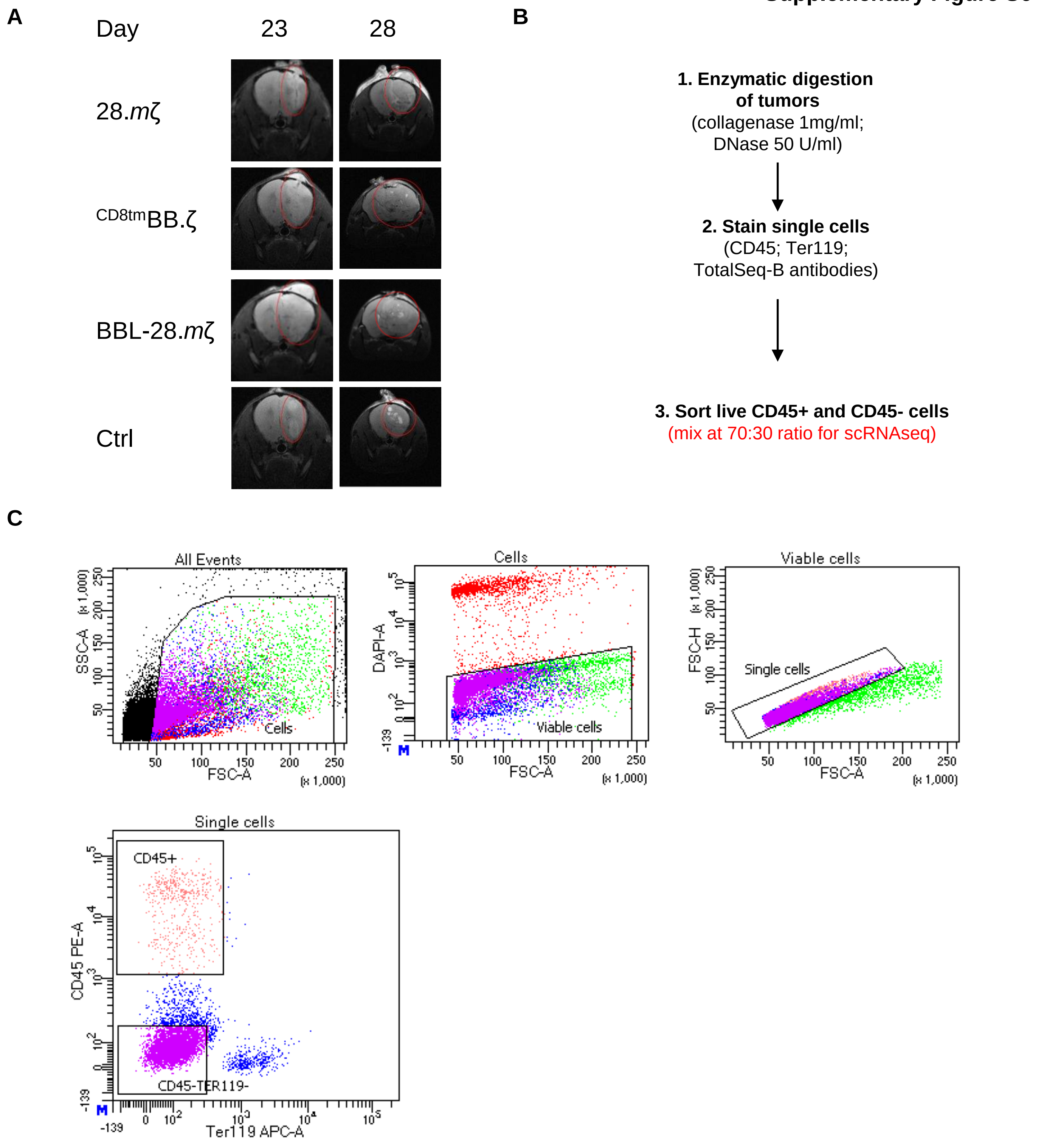

Supplementary Fig. S6: Experimental set-up of single cell RNAseq experiments for gene expression analyses early after CAR T cell injection. **(A)** Representative images from axial brain MRI before CAR T cell treatment at day 23 post-tumor implantation and right before brain tissue harvesting at day 28 post-tumor implantation. **(B)** Experimental scheme for processing brain tissues for single cell RNAseq. **(C)** Representative flow plot from FACS sorting and gating scheme showing the two viable populations sorted for sequencing analysis ((i) Live, TER119-, CD45+ and (ii) Live, TER119-, CD45-). Samples i and ii were mixed at 70:30 ratio prior to loading on chromium controllers.
