# Supplementary material for: CAR T-cell Design-dependent Remodeling of the Brain Tumor Immune Microenvironment Modulates Tumor-associated Macrophages and Anti-glioma Activity
Source: Cancer Res Commun. 2023 Dec 1;3(12):2430–46. doi: 10.1158/2767-9764.CRC-23-0424 (PMC10689147; doi:10.1158/2767-9764.CRC-23-0424)
Supplement: Supplementary Figure 7 — Supplementary Figure S7 shows initial analysis of scRNAseq data. [file crc-23-0424-s09.pdf]

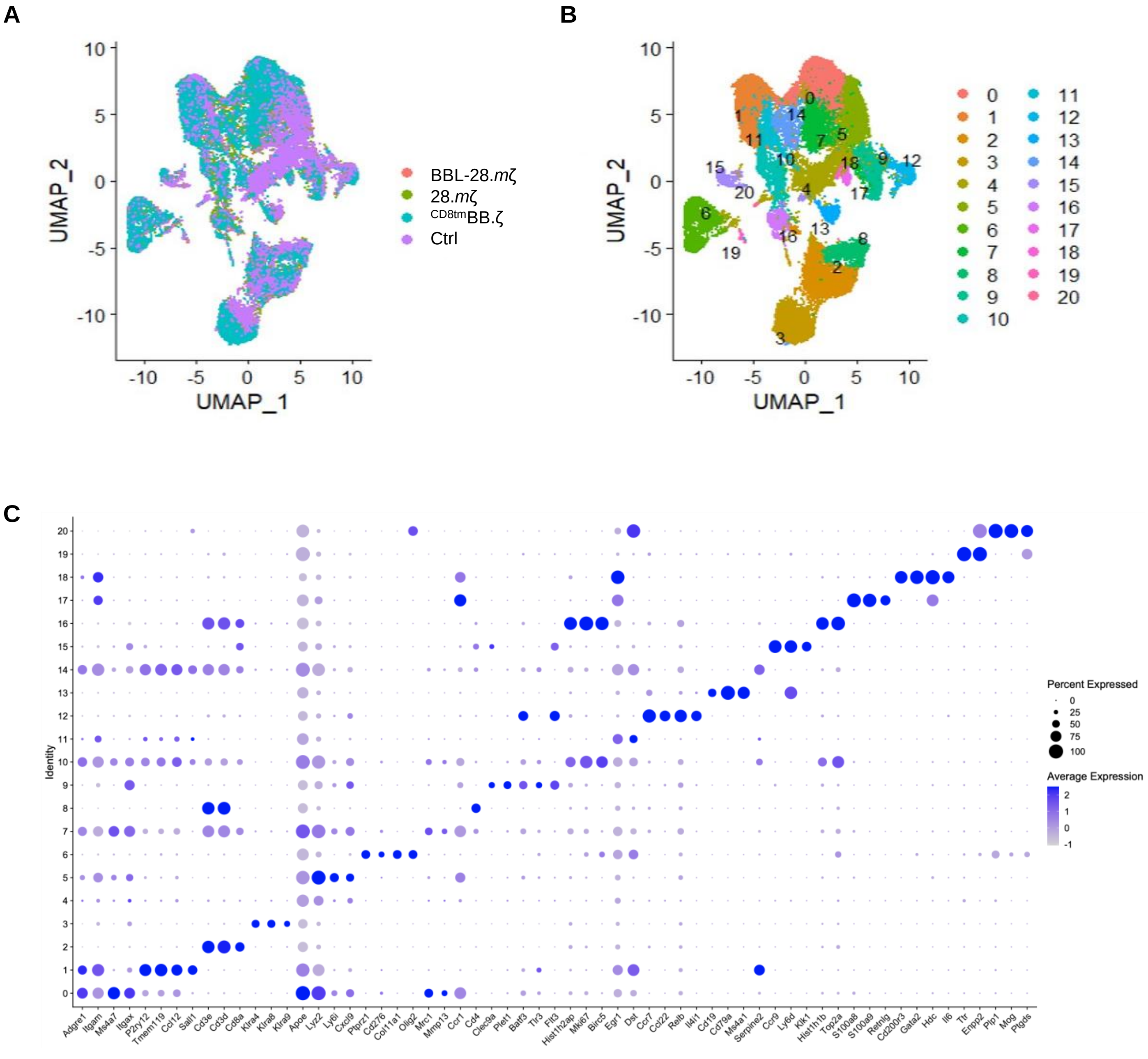

Supplementary Fig. S7: Analysis of scRNAseq data using 10 dimensionality reduction method. **(A)** UMAP visualization of major immune cell clusters per treatment group. **(B)** UMAP plot of single cells from four treatment groups colored by their distribution into 21 major cell types. **(C)** Dot plot visualizing the canonical cell-type marker expression within each cell cluster.
