# Supplementary material for: CAR T-cell Design-dependent Remodeling of the Brain Tumor Immune Microenvironment Modulates Tumor-associated Macrophages and Anti-glioma Activity
Source: Cancer Res Commun. 2023 Dec 1;3(12):2430–46. doi: 10.1158/2767-9764.CRC-23-0424 (PMC10689147; doi:10.1158/2767-9764.CRC-23-0424)
Supplement: Supplementary Figure 8 — Supplementary Figure S8 provides cluster identities. [file crc-23-0424-s10.pdf]

Cluster	Functional group	Subgroup (if any)	Gene Markers
**0**	Mac-1	Monocyte-derived TAM	*Adgre1, Itgam, Itgax, Itga4 (Ms4a7, Pf4)*
**1**	MG-1	Brain Microglia	*P2ry12, Tmem119, Ccl12, Sall1*
**2**	T cells	CD8	*Cd3e, Cd3d, Cd8a*
**3**	NK cells		*klra4, klra8, klra9*
**4**	Other	Myeloid/granulocyte	*Mix*
**5**	Monocytes	Inflammatory	*Ly6i, Cxcl9, Cxcl10, Lyz2*
**6**	Tumor cells		*Ptprz1, Cd276, Col11a1, Olig2*
**7**	Mac-2	Monocyte-derived TAM	*Adgre1, Itgam, Itgax, Itga4 (Mrc1, Mmp13, Ccl8, Ifng)*
**8**	T cells	CD4, Treg	*Cd4, Foxp3*
**9**	DC-1	cDC1	*Xcr1, Clec9a, Plet1, Tlr3, Batf3, Flt3*
**10**	MG/Mac-1	proliferative microglia-derived TAM	*Adgre1, Itgam (Birc5, Hist1h2ap, Mki67)*
**11**	MG-2	Mixed population	*Egr1, Dst, Mef2*
**12**	DC-2	cDC, migratory	*Ccr7, Ccl22, Relb, Il4i1*
**13**	B cells		*Cd79a, Cd19, Ms4a1*
**14**	MG/Mac-2	Inflammatory	*Tmem119, Cxcr1, Serpine2*
**15**	DC-3	pDCs	*Ccr9, Ly6d, Klk1*
**16**	T cells	Proliferating	*Hist1h1b, Top2a*
**17**	Neutrophils		*Ly6g, S100a8, S100a9, Retnlg*
**18**	Basophils		*Cd200r3, Gata2, Il6*
**19**	Epithelial/CNS		*Ttr, Enpp2*
**20**	Fibroblasts	oligodendrocytes	*Plp1, Mog, Ptgds*

Supplementary Fig. S8: Manual validation of immune cell clusters from scRNAseq data. Cluster identities were verified by expression of canonical markers for each cell type as well as by manual review of top upregulated genes relevant to the functional pathways within each cluster.
Mac – macrophages, MG – microglia, DC – dendritic cells, CNS – central nervous system
