# Supplementary material for: CAR T-cell Design-dependent Remodeling of the Brain Tumor Immune Microenvironment Modulates Tumor-associated Macrophages and Anti-glioma Activity
Source: Cancer Res Commun. 2023 Dec 1;3(12):2430–46. doi: 10.1158/2767-9764.CRC-23-0424 (PMC10689147; doi:10.1158/2767-9764.CRC-23-0424)
Supplement: Supplementary Figure 9 — Supplementary Figure S9 shows cluster frequency per treatment group for each cluster. [file crc-23-0424-s11.pdf]

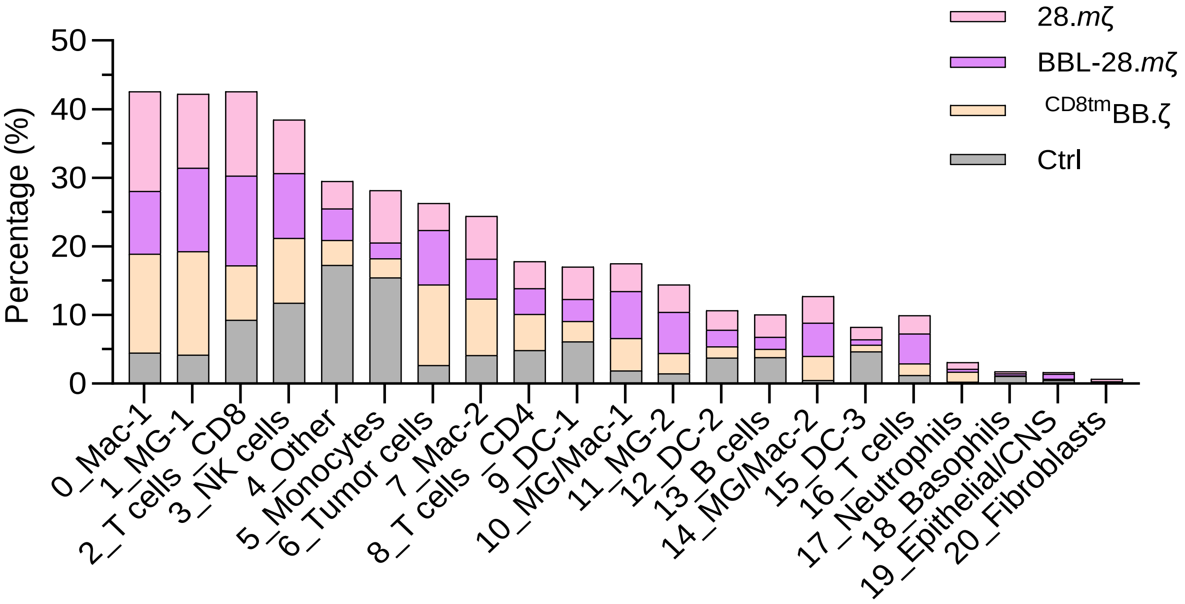

Supplementary Fig. S9: Analysis of TIME composition at 4-days post CAR T-cell treatment. Quantitative plot showing cluster frequency (percentage) per treatment group for each of 21 Seurat clusters in the dataset.
