# Supplementary material for: CAR T-cell Design-dependent Remodeling of the Brain Tumor Immune Microenvironment Modulates Tumor-associated Macrophages and Anti-glioma Activity
Source: Cancer Res Commun. 2023 Dec 1;3(12):2430–46. doi: 10.1158/2767-9764.CRC-23-0424 (PMC10689147; doi:10.1158/2767-9764.CRC-23-0424)
Supplement: Supplementary Figure 10 — Supplementary Figure S10 shows diversity of T cell clusters per treatment group. [file crc-23-0424-s12.pdf]

Supplementary Figure S10

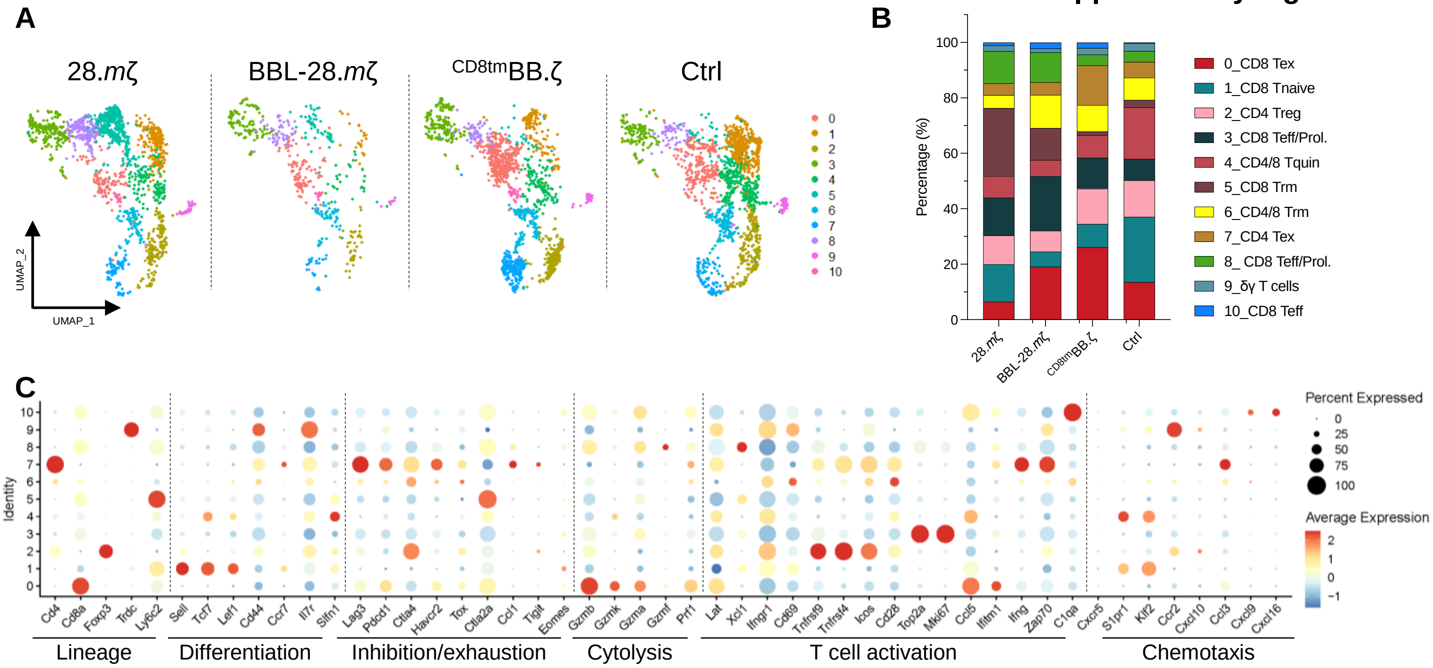

D

T cell cluster	Subgroup	Gene markers
**0**	Activated/Exhausted CD8 Teff	*Gzma, Nkg7, Klrc1, Prf1, Lag3, Ctla4, Havcr2, Pdcd1, Tox*
**1**	CD8 Tnaive	*Sell, Tcf7, Lef1*
**2**	CD4 Treg	*Foxp3, Tnfrsf4, Il2ra*
**3**	Proliferating CD8 Teff	*Hist1h2ap, Mki67, Top2a*
**4**	Resting/quiescent T cells (CD4/8 mix)	*Klf2, Tcf7, Slfn1, Klf3*
**5**	CD8 TRM	*Ly6c2, Xcl1, Cxcr6*
**6**	Activated/Exhausted TRM (CD4/8 mix)	*Cd69, Cd28, C1qa, Lag3, Ctla4, Havcr2, Tigit*
**7**	Exhausted CD4 T cells	*Lag3, Ctla4, Havcr2, Pdcd1, Tox*
**8**	Proliferating CD8 Teff	*Mcm5, Mcm6, Mcm7, Pcna, Ybx3*
**9**	Gamma-delta T cells	*Trdc, Il17a, Tcrg-C1*
**10**	Activated CD8 Teff	*C1qa, C1rc, H2-Eb1, Cxcl9*

Supplementary Fig. S10: Diversity of endogenous T-cell responses within the glioma TIME post CAR T-cell treatment. Seurat clusters 2, 8, and 16 were reclustered to further analyze lymphoid responses post CAR T-cell treatment. **(A)** UMAP plots of the T-cell subclusters visualized by treatment group. **(B)** Summary plot of T-cell subcluster distribution per treatment. **(C)** Dot plot depicting T-cell lineage and differentiation markers, T-cell immune inhibitory genes, T-cell cytolysis and immune activation genes, and chemotaxis genes per T-cell subcluster. Dot size represents the percentage of cells expressing each gene and dot color represents mean expression level with a gradient of lowest expression in blue to highest expression in red. **(D)** Table showing top upregulated genes corresponding with the functional phenotype for each of the 10 T-cell subclusters.

Tex – Exhausted T-cells, Treg – Regulatory T-cells, Teff– Effector memory T-cells, Tquin – Quinescent T-cells, Trm – Tissue resident memory T-cells, Prol. – Proliferating.
