# Supplementary material for: CAR T-cell Design-dependent Remodeling of the Brain Tumor Immune Microenvironment Modulates Tumor-associated Macrophages and Anti-glioma Activity
Source: Cancer Res Commun. 2023 Dec 1;3(12):2430–46. doi: 10.1158/2767-9764.CRC-23-0424 (PMC10689147; doi:10.1158/2767-9764.CRC-23-0424)
Supplement: Supplementary Figure 11 — Supplementary Figure S11 shows dot plots depicting differentially expressed T cell genes in T cell clusters per treatment group. [file crc-23-0424-s13.pdf]

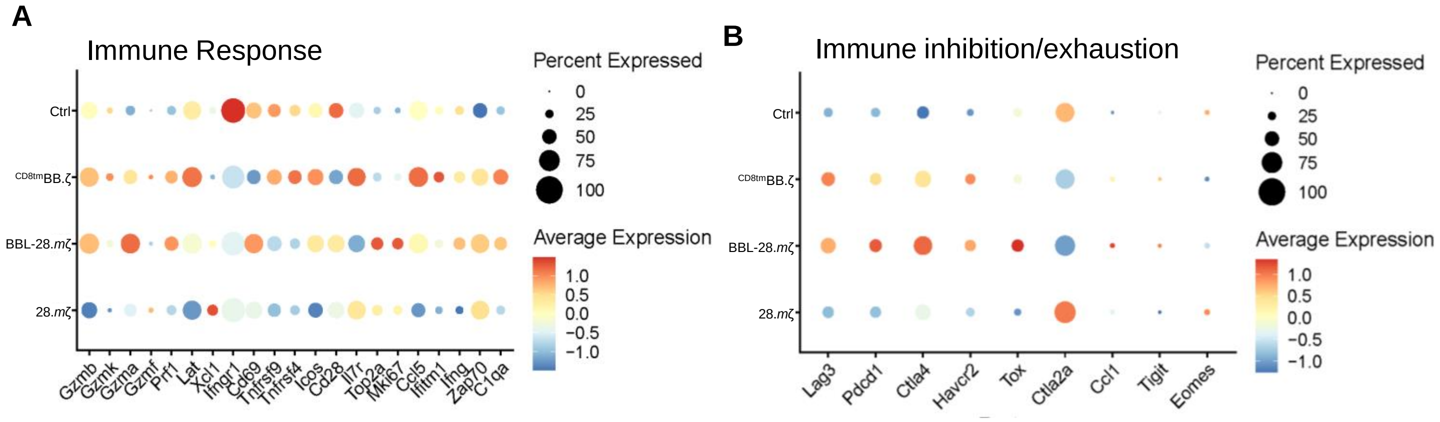

Supplementary Fig. S11: Heterogeneity analysis of T-cell responses post CAR T-cell treatment in GL261 glioma bearing mice. **(A-B)** Dot plots depicting differentially expressed genes associated with T-cell immune activation **(A)** and inhibition/exhaustion **(B)** per treatment group.
