# Supplementary material for: CAR T-cell Design-dependent Remodeling of the Brain Tumor Immune Microenvironment Modulates Tumor-associated Macrophages and Anti-glioma Activity
Source: Cancer Res Commun. 2023 Dec 1;3(12):2430–46. doi: 10.1158/2767-9764.CRC-23-0424 (PMC10689147; doi:10.1158/2767-9764.CRC-23-0424)
Supplement: Supplementary Figure 12 — Supplementary Figure S12 shows CAR presence in each cluster and GSEA analysis in specific clusters. [file crc-23-0424-s14.pdf]

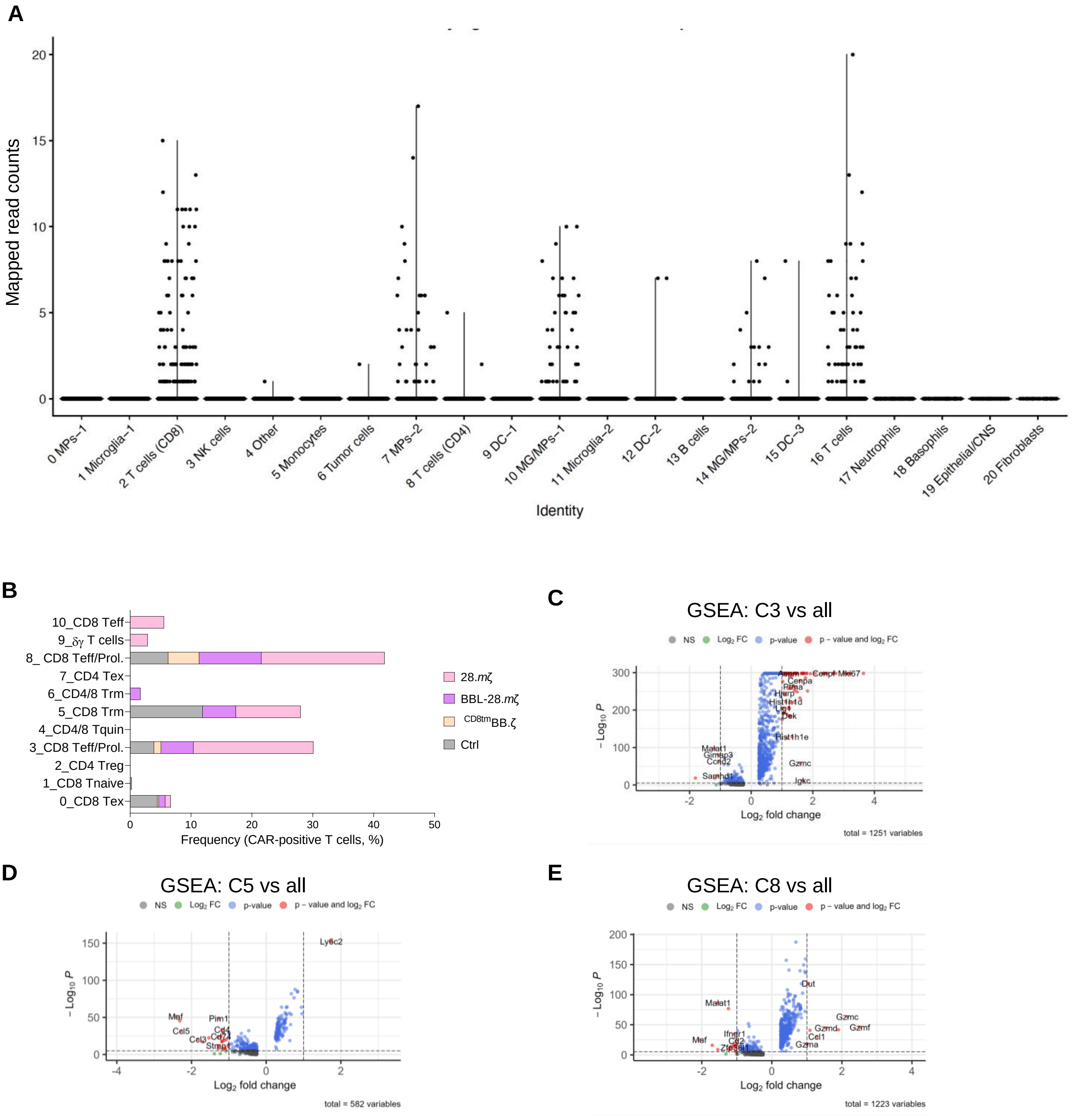

Supplementary Fig. S12: Detection and characterization of CAR T cells within the glioma TIME. **(A)** Mapped read counts for CAR scFv sequences in each of the 21 Seurat clusters (MP – macrophages, MG – microglia, DC – dendritic cells). **(B)** Summary plot showing expression of CAR molecules per T-cell subclusters and colored per treatment group within each cluster. **(C-E)** Volcano plots showing differentially –up and –down regulated genes in T-cell subclusters C3 in **(C)**, C5 in **(D)**, and C8 in **(E)** as compared to all other T-cell subclusters.
