# Supplementary material for: CAR T-cell Design-dependent Remodeling of the Brain Tumor Immune Microenvironment Modulates Tumor-associated Macrophages and Anti-glioma Activity
Source: Cancer Res Commun. 2023 Dec 1;3(12):2430–46. doi: 10.1158/2767-9764.CRC-23-0424 (PMC10689147; doi:10.1158/2767-9764.CRC-23-0424)
Supplement: Supplementary Figure 13 — Supplementary Figure S13 shows cell-cell communication analysis within the TIME from our scRNAseq dataset. [file crc-23-0424-s15.pdf]

Supplementary Figure S13

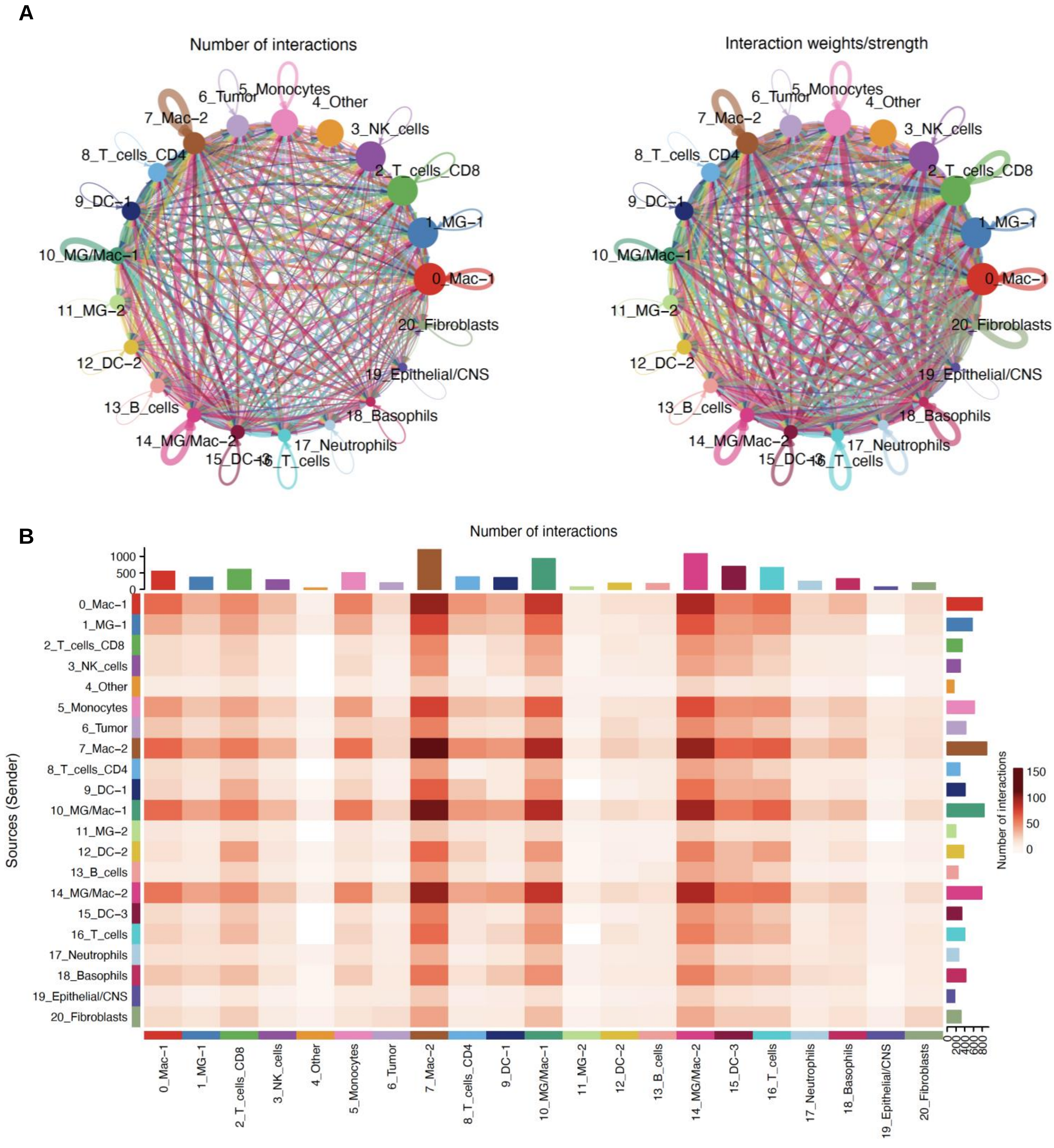

Supplementary Fig. S13: Analysis of cell-cell communication within the TIME from our scRNAseq dataset. The signaling between each of the 21 Seurat clusters were analyzed and reconstructed with CellChat. **(A)** Circle plot showing number and intensity of significant ligand-receptor interactions between any two pairs of Seurat clusters. **(B)** Heatmap quantifies the role of each cluster as a sender and receiver/mediator of each interaction.

Mac – macrophages, MG – microglia, DC – dendritic cells.
