# Supplementary material for: CAR T-cell Design-dependent Remodeling of the Brain Tumor Immune Microenvironment Modulates Tumor-associated Macrophages and Anti-glioma Activity
Source: Cancer Res Commun. 2023 Dec 1;3(12):2430–46. doi: 10.1158/2767-9764.CRC-23-0424 (PMC10689147; doi:10.1158/2767-9764.CRC-23-0424)
Supplement: Supplementary Figure 14 — Supplementary Figure S14 shows macrophage populations post CAR T-cell treatment in GL261 glioma bearing mice. [file crc-23-0424-s16.pdf]

A

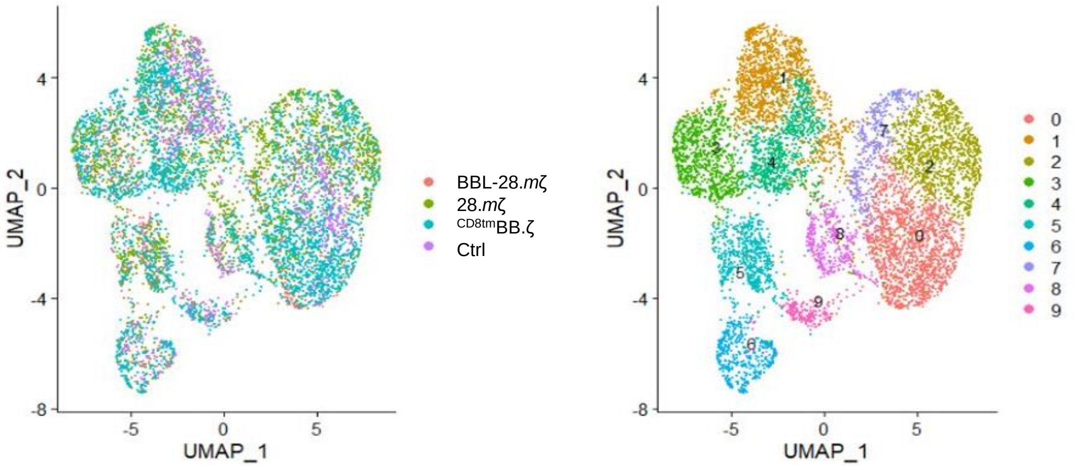

B

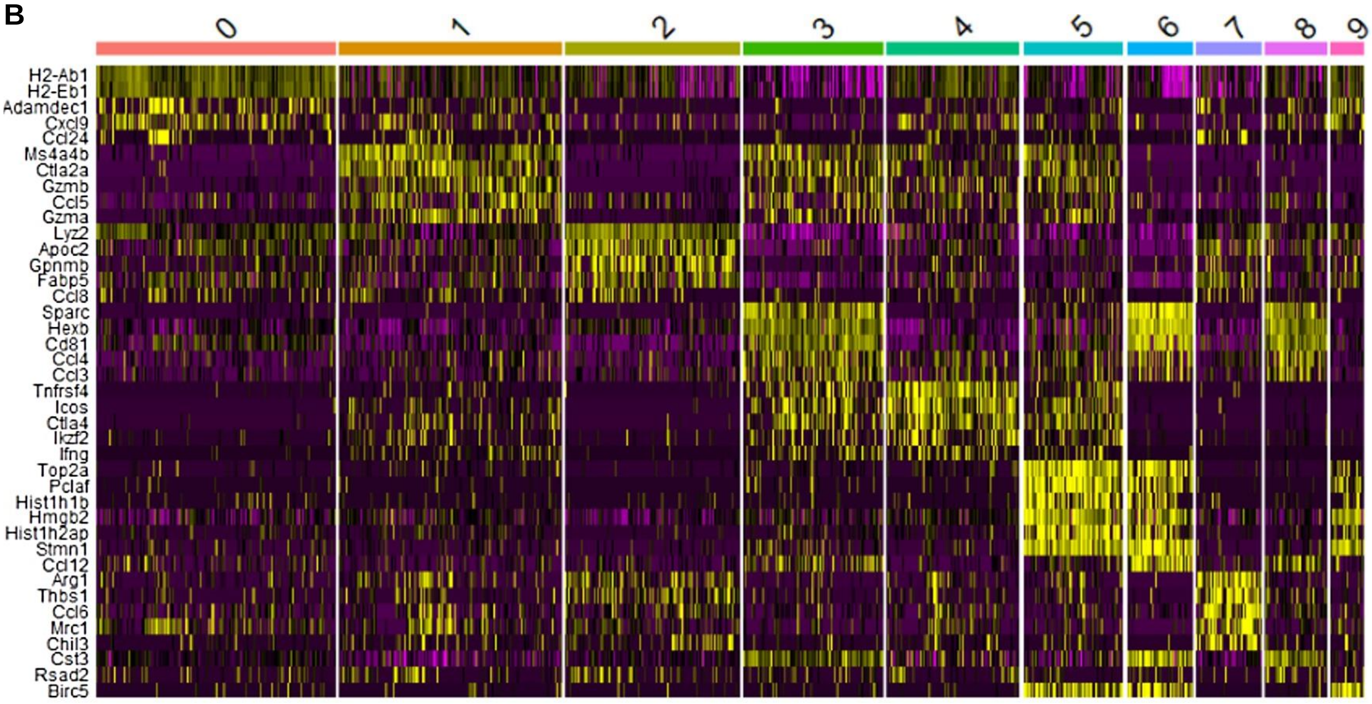

C

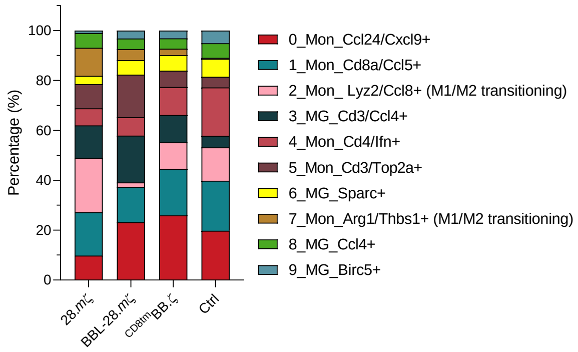

Supplementary Fig. S14: Heterogeneity analysis of macrophage populations post CAR T-cell treatment in GL261 glioma bearing mice. Clusters 0, 7, 10, and 14 were re-clustered to further define macrophage populations across treatment groups. **(A)** UMAP plot of macrophage subclusters visualized by color according to each treatment group on the left and by each of the 10 major macrophage clusters across the four treatment groups on the right. **(B)** Heatmap showing top upregulated genes within each of the 10 macrophage subclusters. **(C)** Summary plot of macrophage subcluster distribution per treatment.
