# Supplementary material for: CAR T-cell Design-dependent Remodeling of the Brain Tumor Immune Microenvironment Modulates Tumor-associated Macrophages and Anti-glioma Activity
Source: Cancer Res Commun. 2023 Dec 1;3(12):2430–46. doi: 10.1158/2767-9764.CRC-23-0424 (PMC10689147; doi:10.1158/2767-9764.CRC-23-0424)
Supplement: Supplementary Figure 15 — Supplementary Figure S15 shows GSEA of hallmark pathways in macrophage subclusters. [file crc-23-0424-s17.pdf]

Supplementary Figure S15

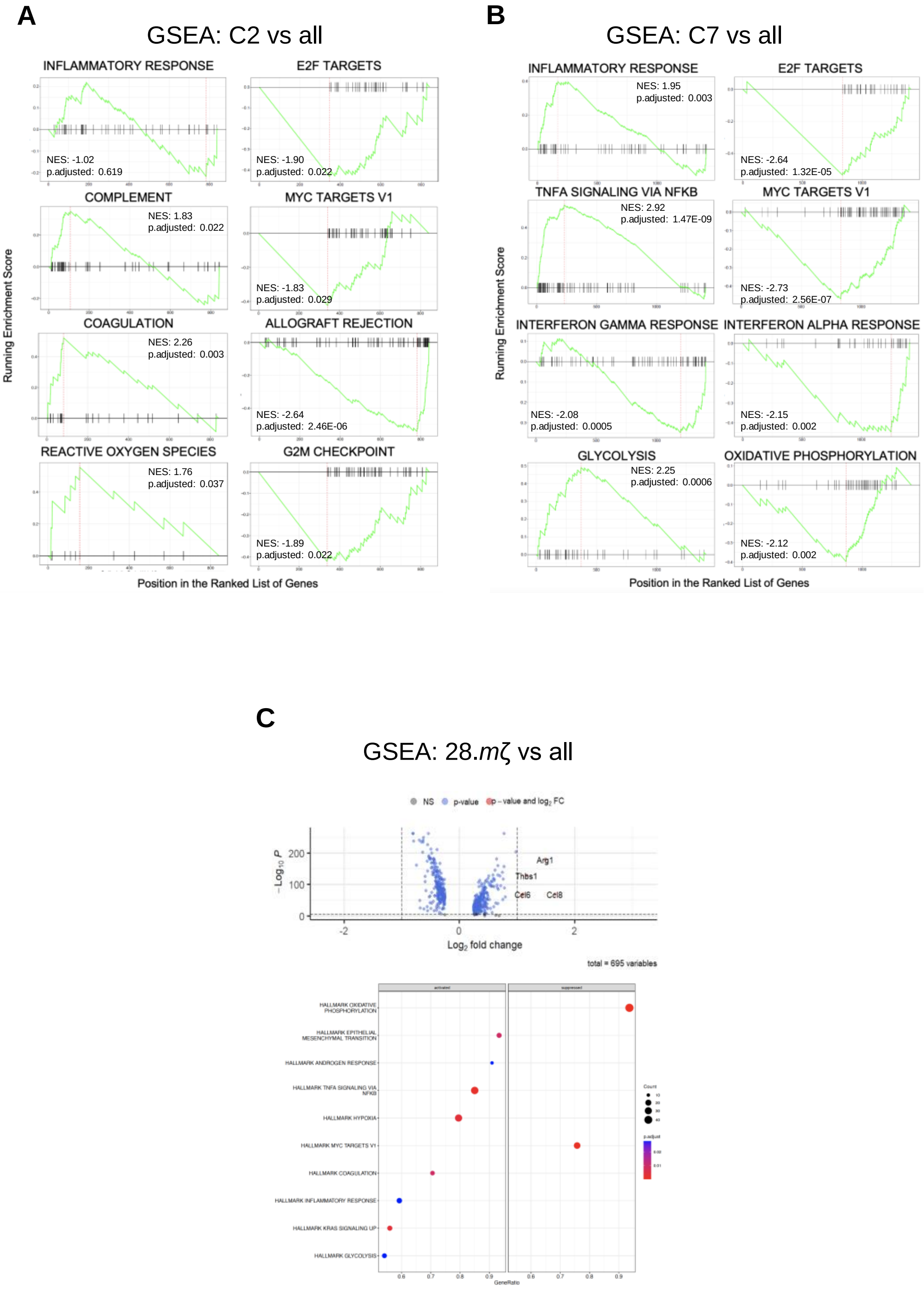

Supplementary Fig. S15: Gene set enrichment analysis (GSEA) of hallmark pathways differentially up- or down- regulated in macrophage subclusters. **(A)** Enrichment plots of top six hallmark pathways involved in pro- and anti- inflammatory macrophage functions from GSEA Hallmark analysis comparing macrophage subcluster 2 versus other macrophage subclusters. Ranked genes depicted on the x-axis with a black line with most enriched on the left to least enriched on the right. Normalized enrichment score (NES) depicted as well as adjusted p-value (p. adjusted). **(B)** Enrichment plots for macrophage hallmark pathways enriched in subcluster 7 as compared to other macrophage subclusters. **(C)** Volcano plot showing differential gene expression profiles in macrophage subclusters associated with 28.*mζ*-CAR treatment compared to all other groups along with dot plot for top 10 differentially up- or down- regulated hallmark pathways in macrophage subclusters associated with 28.*mζ*-CAR treatment compared to other CAR groups.
