# Supplementary material for: CAR T-cell Design-dependent Remodeling of the Brain Tumor Immune Microenvironment Modulates Tumor-associated Macrophages and Anti-glioma Activity
Source: Cancer Res Commun. 2023 Dec 1;3(12):2430–46. doi: 10.1158/2767-9764.CRC-23-0424 (PMC10689147; doi:10.1158/2767-9764.CRC-23-0424)
Supplement: Supplementary Figure 16 — Supplementary Figure S16 shows intracellular flow cytometry data from the endpoint tumors after CAR T cell treatment. [file crc-23-0424-s18.pdf]

Supplementary Figure S16

A

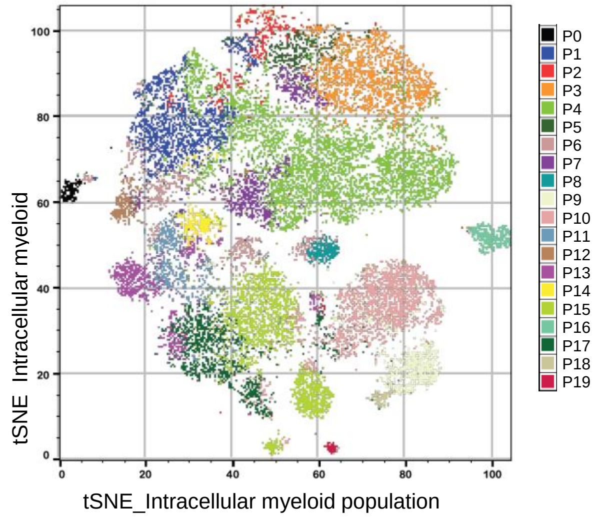

B

Gate: Cell-Sized –> Viable –> Single Cell –> CD45(+) –> CD4(-) –> CD8(-) –> B220(-)

Cluster	Count	ARG1	IL17a	CD11c	IFNg	RORgt	CD25	CD11b	TNFa	F4/80	CD90.1	IL6	AHR	IL10	FoxP3
0	199														
1	1731														
2	468														
3	2184														
4	5939														
5	528														
6	921														
7	1101														
8	383														
9	951														
10	2183														
11	804														
12	369														
13	983														
14	468														
15	2541														
16	450														
17	1518														
18	170														
19	109														

C

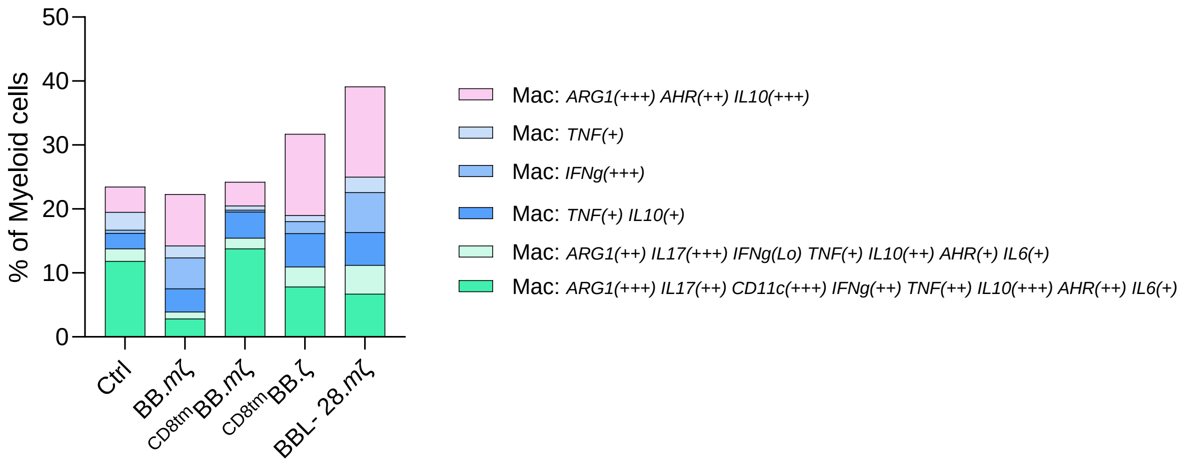

Supplementary Fig. S16: Unsupervised nonlinear dimensionality reduction of intracellular flow cytometry data from tumors at endpoint. Tumors were permeabilized, fixed, and processed for intracellular staining. Detailed information about surface and intracellular marker expression patterns in the tumor infiltrates was obtained after t-SNE algorithms were applied with the aid of FlowJo software (Becton Dickenson, San Jose). The identity of each separate cluster in the t-SNE plot was confirmed and further visualized using a FlowSOM heatmap. **(A)** Representative cluster plot of t-SNE mapping for myeloid cell subsets stained for intracellular markers along with the corresponding FlowSOM heatmap for individual markers in **(B)**. **(C)** Summary plot of the macrophage/microglia clusters based on their expression of specific intracellular markers. The number of + signs increases with increased MFI for each marker in the relevant cell cluster.
