# Supplementary material for: CAR T-cell Design-dependent Remodeling of the Brain Tumor Immune Microenvironment Modulates Tumor-associated Macrophages and Anti-glioma Activity
Source: Cancer Res Commun. 2023 Dec 1;3(12):2430–46. doi: 10.1158/2767-9764.CRC-23-0424 (PMC10689147; doi:10.1158/2767-9764.CRC-23-0424)
Supplement: Supplementary Figure 17 — Supplementary Figure S17 shows testing of BLZ945 effect on CAR T cell in vitro. [file crc-23-0424-s19.pdf]

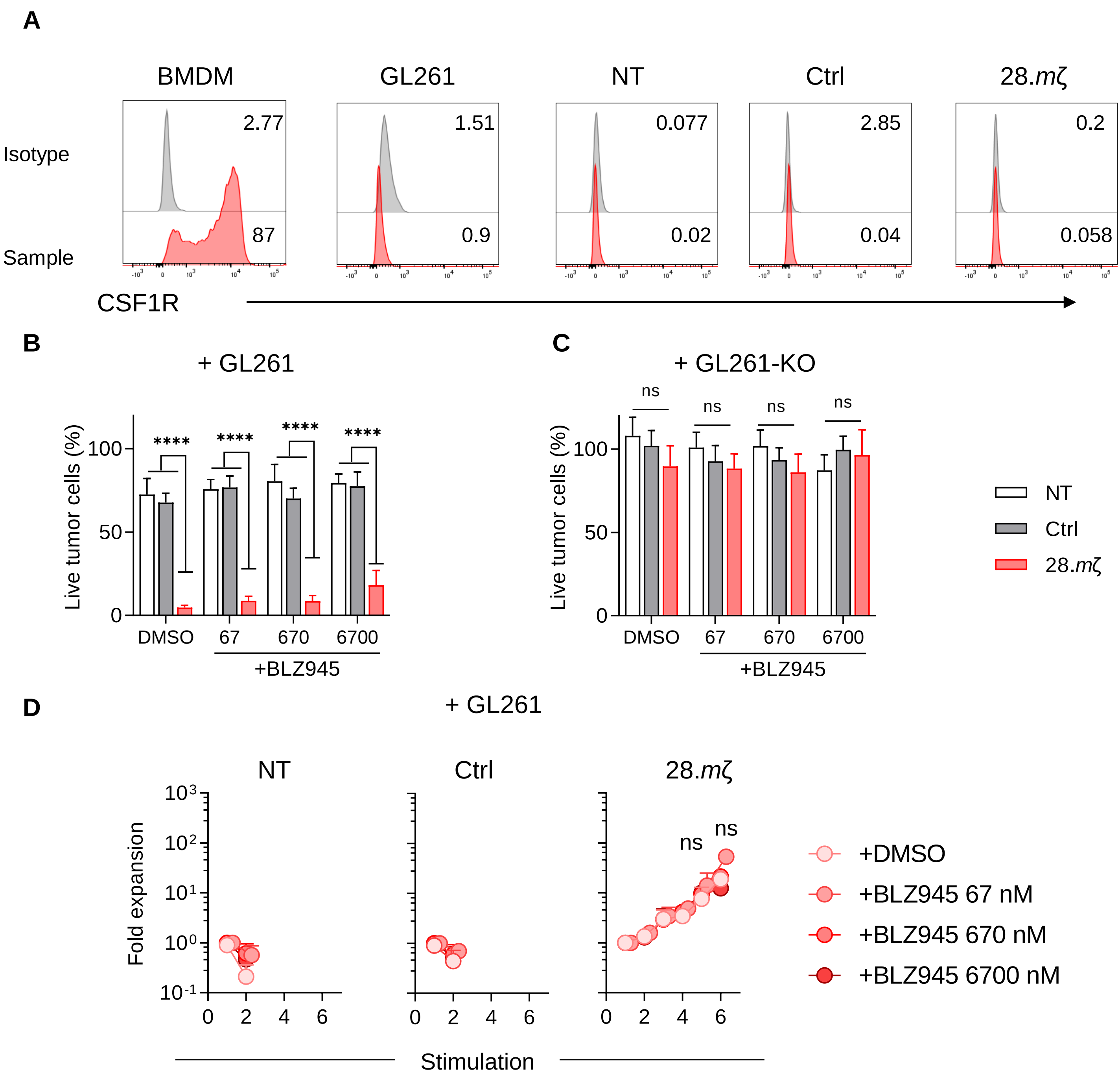

Supplementary Fig. S17: CSF1R inhibition does not affect B7-H3 CAR T-cell functions *in vitro.* **(A)** Representative flow plots showing CSF1R expression on bone marrow derived macrophages (BMDM), Gl261 tumor cells, NT T-cells, as well as Ctrl and B7-H3 CAR T-cells. **(B-C)** MTS cytotoxicity assay at an effector to target (E:T) ratio of 0.25:1 against GL261 tumor cells in **(B)** and GL261 *B7h3*-KO tumor cells in **(C)** in the presence of BLZ945 at different concentrations (n = 4, mean ± SD, 2-way ANOVA with Tukey's test for multiple comparisons). **(D)** NT, Ctrl and B7-H3 CAR T-cells were cocultured with GL261 tumor cells at a 2:1 ratio in the presence of BLZ945 at different concentrations. T-cells were restimulated every 3-days against fresh tumor cells until they no longer killed and/or expanded. Graphs depict fold expansion of T-cells upon successive stimulations (x-axis: each stimulation is a 3-day co-culture with fresh GL261 tumor cells, n = 6).
