# Supplementary material for: CAR T-cell Design-dependent Remodeling of the Brain Tumor Immune Microenvironment Modulates Tumor-associated Macrophages and Anti-glioma Activity
Source: Cancer Res Commun. 2023 Dec 1;3(12):2430–46. doi: 10.1158/2767-9764.CRC-23-0424 (PMC10689147; doi:10.1158/2767-9764.CRC-23-0424)
Supplement: Supplementary Figure 18 — Supplementary Figure S18 shows BLZ945 effect on CAR T cells in media and in co-culture with GL261-KO cells. [file crc-23-0424-s20.pdf]

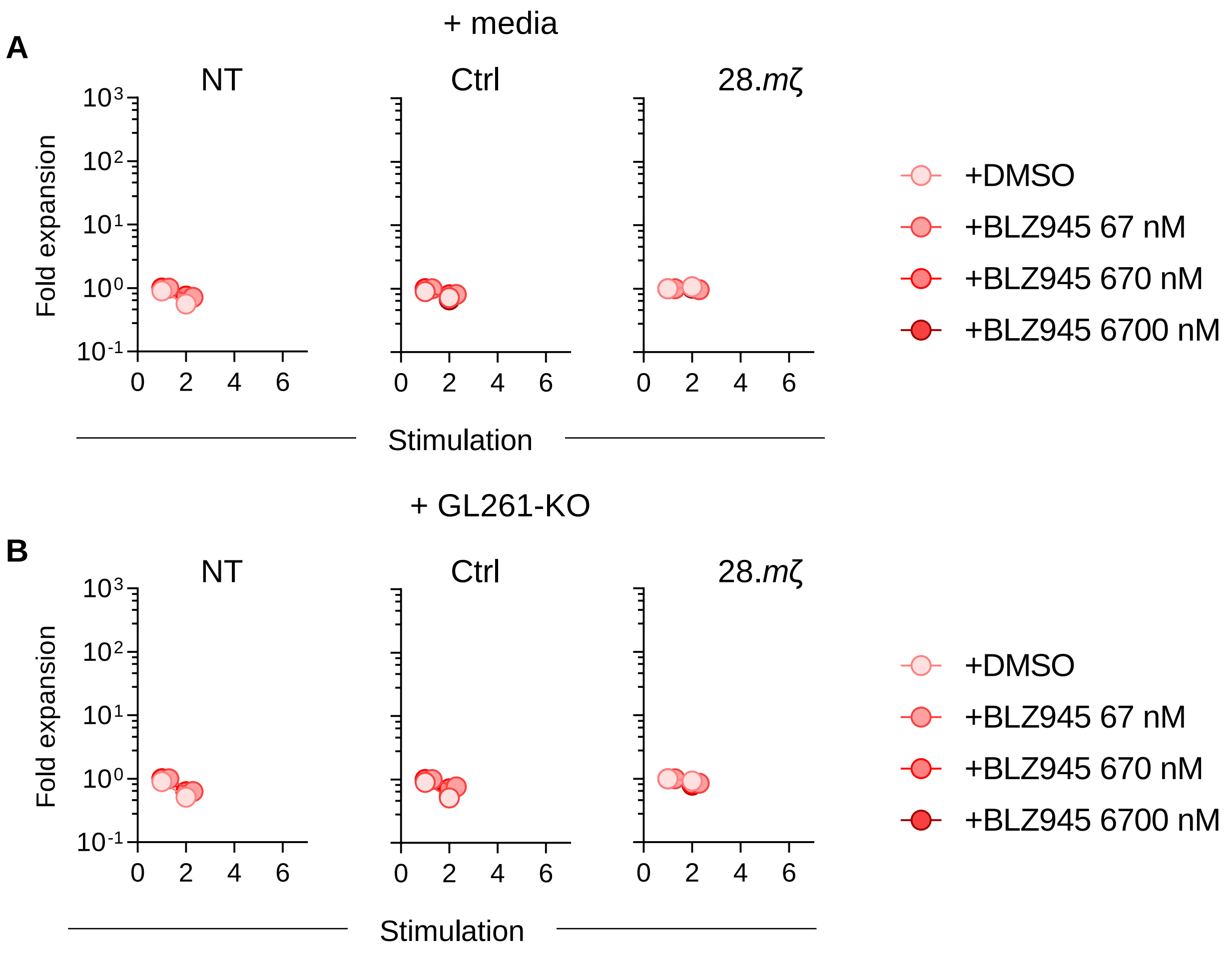

Supplementary Fig. S18: CSF1R inhibition does not induce non-specific killing with B7H3 CAR T-cells *in vitro*. NT, Ctrl and B7-H3 CAR T-cells were cocultured with media only or GL261 *B7h3*-KO tumor cells at a 2:1 ratio in the presence of BLZ945 at different concentrations with restimulation every 3-days against fresh tumor cells until T-cells no longer killed and/or expanded. **(A)** Fold expansion of CAR T-cells in the absence of target cells at different BLZ945 concentrations. **(B)** Expansion of CAR T-cells upon stimulation with antigen-negative GL261 *B7h3*-KO tumor cells with different BLZ945 concentrations (n = 5).
