# Supplementary material for: CAR T-cell Design-dependent Remodeling of the Brain Tumor Immune Microenvironment Modulates Tumor-associated Macrophages and Anti-glioma Activity
Source: Cancer Res Commun. 2023 Dec 1;3(12):2430–46. doi: 10.1158/2767-9764.CRC-23-0424 (PMC10689147; doi:10.1158/2767-9764.CRC-23-0424)
Supplement: Supplementary Figure 19 — Supplementary Figure S19 shows IHC for F4/80 and CD11c after BLZ945 and CAR T cell treatment, and OS post combination treatment. [file crc-23-0424-s21.pdf]

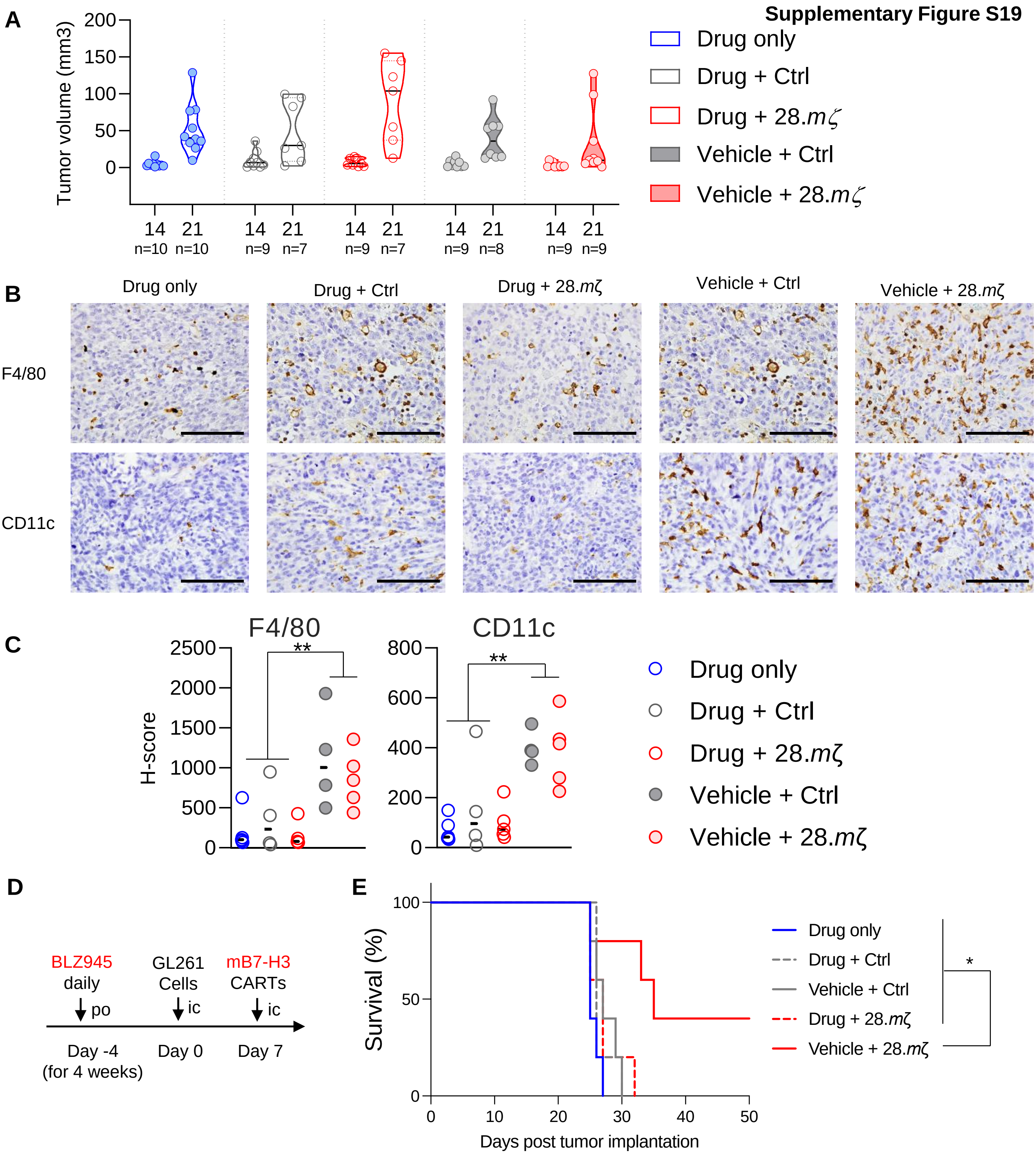

Supplementary Fig. S19: CSF1R inhibition abrogates anti-glioma efficacy of B7H3 CAR T-cells. Gl261 glioma-bearing mice were treated with BLZ945 at 200mg/Kg followed by CAR T-cell injection intratumorally. **(A)** Tumor volumes determined from MRI in mice treated with BLZ945 (drug) in combination with Ctrl or B7-H3 CAR T-cells. **(B)** Representative pictures from immunostaining of F4/80 and CD11c from mice treated with BLZ945 combination with B7H3 CAR T-cells at 40x magnification (scale bar = 100 μm). **(C)** Summary plot of F4/80 and CD11c quantitative analysis as evaluated by blinded pathologist ($P<0.001$). **(D)** Experimental scheme of BLZ945 treatment in combination with B7-H3 CAR T-cells starting with daily drug dosing 4-days prior to tumor implantation followed by CAR T-cell treatment at day-7 post implantation. **(E)** Kaplan-Meier survival curve. N=5. $P<0.05$
